# *Mycobacterium tuberculosis* WhiB4 regulates oxidative stress response to modulate survival and dissemination *in vivo*

**DOI:** 10.1111/j.1365-2958.2012.08165.x

**Published:** 2012-07-26

**Authors:** Manbeena Chawla, Pankti Parikh, Alka Saxena, MohamedHusen Munshi, Mansi Mehta, Deborah Mai, Anup K Srivastava, K V Narasimhulu, Kevin E Redding, Nimi Vashi, Dhiraj Kumar, Adrie J C Steyn, Amit Singh

**Affiliations:** 1 Immunology Group, International Centre for Genetic Engineering and BiotechnologyNew Delhi 110067, India; 2 Department of Microbiology, University of Alabama at BirminghamBirmingham, AL 35294, USA; 3 Pediatric Hematology & Oncology Research, St. Joseph's Children's Hospital TampaFL 33607, USA; 4 Department of Chemistry and Biochemistry, Arizona State UniversityAZ 85287, USA; 5 KwaZulu-Natal Research Institute for Tuberculosis and HIV (K-RITH)Durban 4001, South Africa

## Abstract

Host-generated oxidative stress is considered one of the main mechanisms constraining *Mycobacterium tuberculosis* (*Mtb*) growth. The redox-sensing mechanisms in *Mtb* are not completely understood. Here we show that WhiB4 responds to oxygen (O_2_) and nitric oxide (NO) via its 4Fe-4S cluster and controls the oxidative stress response in *Mtb*. The WhiB4 mutant (*Mtb*Δ*whiB4*) displayed an altered redox balance and a reduced membrane potential. Microarray analysis demonstrated that *Mtb*Δ*whiB4* overexpresses the antioxidant systems including alkyl hydroperoxidase (*ahpC-ahpD*) and rubredoxins (*rubA-rubB*). DNA binding assays showed that WhiB4 [4Fe-4S] cluster is dispensable for DNA binding. However, oxidation of the apo-WhiB4 Cys thiols induced disulphide-linked oligomerization, DNA binding and transcriptional repression, whereas reduction reversed the effect. Furthermore, WhiB4 binds DNA with a preference for GC-rich sequences. Expression analysis showed that oxidative stress repressed *whiB4* and induced antioxidants in *Mtb*, while their hyper-induction was observed in *Mtb*Δ*whiB4*. *Mtb*Δ*whiB4* showed increased resistance to oxidative stress *in vitro* and enhanced survival inside the macrophages. Lastly, *Mtb*Δ*whiB4* displayed hypervirulence in the lungs of guinea pigs, but showed a defect in dissemination to their spleen. These findings suggest that WhiB4 systematically calibrates the activation of oxidative stress response in *Mtb* to maintain redox balance, and to modulate virulence.

## Introduction

The success of *Mtb* as a human pathogen hinges on its ability to latently infect ∼ 2 billion people worldwide and then potentially reactivating in a subset of infected individuals. Molecular mechanisms underlying *Mtb* persistence and reactivation remain poorly understood, but are critical towards the development of novel strategies against tuberculosis (TB) infection. The production of reactive oxygen and nitrogen intermediates (ROI and RNI respectively) by macrophages is considered to be the major mechanism restraining *Mtb* proliferation *in vivo* (MacMicking *et al*., 1997; Ng *et al*., 2004). ROI and RNI generate intracellular redox stress and kill bacteria by damaging biomolecules such as DNA, proteins and lipids. Studies have shown that nitric oxide synthase-2 (NOS2) and phagocyte oxidase (PHOX) gene knockout mice are defective in generating RNI and ROI, respectively, and exhibit increased sensitivity to *Mtb* infection (MacMicking *et al*., 1997; Cooper *et al*., 2000). The importance of ROI in controlling *Mtb* infection in humans came from the observation that children with defective oxidative burst mechanisms are highly susceptible to TB and develop severe complications from BCG vaccination (Lee *et al*., 2008). Despite these redox-based bactericidal stresses, *Mtb* can persist for decades in a non-replicative state *in vivo*. These studies suggest that *Mtb* genes involved in resistance to oxidative or nitrosative stress would play a role in persistence. However, the underlying mechanism of how oxidative and nitrosative stress is sensed by *Mtb* to co-ordinate the expression of virulence genes for persistence is poorly understood.

Thiol and/or Fe-S cluster-based transcription factors such as OxyR and SoxR are known to sense oxidative and/or nitrosative stress in bacteria. OxyR responds to peroxide stress by a thiol-disulphide redox switch and controls the transcription of antioxidant systems such as catalase (KatG) and alkyl hydroperoxide reductase (AhpCF) (Green and Paget, 2004). SoxR regulates the expression of a large number of stress-responsive genes by sensing nitrosative and oxidative stress via its redox-responsive 2Fe-2S cluster (Green and Paget, 2004). OxyR and SoxR homologues are found in many bacterial species. However a prototypical homologue of SoxR is absent in the *Mtb* genome, and OxyR is non-functional due to the presence of multiple mutations in its open reading frame (ORF) (Deretic *et al*., 1995). The absence of these regulators in *Mtb* is very intriguing and indicates that *Mtb* might possess novel redox-sensing proteins to control its survival in response to ROI/RNI stress during infection.

Recent studies suggest that *Mtb* is capable of sensing redox signals, such as O_2_ and NO, via the haem-based DosR/S/T system, thiol-based SigH/RshA system, in addition to the family of Fe-S cluster-containing WhiB proteins (den Hengst and Buttner, 2008). The WhiB proteins are putative transcription factors that have been shown to regulate diverse functions, including pathogenesis, cell division, oxidative stress, nitrosative stress, reductive stress, disulphide reductase and antibiotic resistance (Farhana *et al*., 2010). These studies suggest that WhiB proteins might have a functional role in *Mtb* that is similar to OxyR and SoxR in other bacteria. Several studies have indicated an important function of WhiB4 in the pathophysiology of *Mtb*. For example, WhiB4 is induced during long-term hypoxia (Rustad *et al*., 2008) and upon nutrient starvation (Betts *et al*., 2002). WhiB4 expression is also influenced by oxidants [diamide and cumene hydroperoxide (CHP)], SDS, ethanol, isoniazid (INH) and inside macrophages (Geiman *et al*., 2006; Rachman *et al*., 2006). However, the molecular function of WhiB4 in *Mtb* remains uncharacterized.

In this study, we comprehensively analysed the redox state and the O_2_- and NO-sensing properties of the WhiB4 Fe-S cluster using low temperature EPR. We performed global microarray analysis to identify genes controlled by WhiB4. We systematically analysed the capacity of WhiB4 to bind to the promoter regions of antioxidant genes in a redox-dependent manner, examined the sequence preference for WhiB4 DNA binding, and investigated the effect of DNA binding on transcription. Lastly, we examined the ability of *Mtb*Δ*whiB4* to survive oxidative stress *in vitro*, inside macrophages and during infection of guinea pigs. Collectively, our findings provide a fresh mechanistic insight into how *Mtb* responds to host generated redox stress via WhiB4 for survival *in vivo*.

## Results

### WhiB4 contains a redox-responsive [4Fe-4S] cluster as a cofactor

**Fig. 1:**
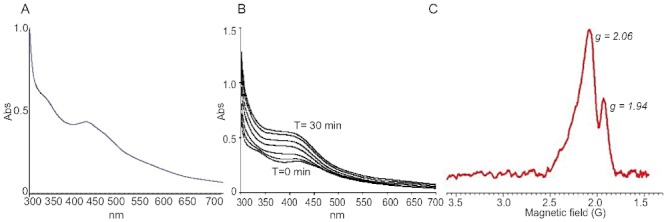
Spectroscopic characterization of WhiB4. A. Aerobically purified WhiB4 was scanned by a UV-vis spectrophotometer. Note the presence of broad peaks at ∼ 330 nm, ∼ 420 and ∼ 450 nm characteristic of a 2Fe-2S cluster. B. UV-visible spectra of anaerobically reconstituted WhiB4. Enzymatic reconstitution of WhiB4 Fe-S was carried out inside an anaerobic glove box*.* Note the time-dependent increase in the characteristic 4Fe-4S cluster peak at 420 nm. Reconstitution of the 4Fe-4S cluster was completed in 30 min. C. EDFS-EPR spectra of reconstituted WhiB4 after reduction with sodium dithionite (DTH). The experimental conditions were: π/2 and π pulses of 16 and 32 ns; τ = 180 ns; T = 9 K. Spectra were acquired 60 shots with a two-step cycle at a repetition rate of 1 kHz. Microwave frequency = 9.806 GHz.

A hexahistidine-tagged version of WhiB4 was expressed in *Escherichia coli* and purified from the soluble fraction by immobilized-metal affinity chromatography. Purified WhiB4 was light brown in colour and displayed broad visible absorption maxima at approximately 330, 420 and 450 nm (Fig. 1A), which are characteristics of proteins containing bacterial type 2Fe-2S ferredoxins (Ta and Vickery, 1992). Since Fe-S clusters are sensitive to degradation during aerobic purification, we reconstituted the Fe-S cluster of WhiB4 under anaerobic conditions using the NifS-catalysed procedure as reported previously (Singh *et al*., 2007). During anaerobic reconstitution of WhiB4 Fe-S cluster, we observed a yellowish brown product along with a time-dependent increase in the absorption intensity at ∼ 420 nm with no other resolved features (Fig. 1B). These spectral features are consistent with the presence of a FNR-type 4Fe-4S cluster in WhiB4 (Khoroshilova *et al*., 1997).

Next, we examined the redox state of the 4Fe-4S cluster in WhiB4 by echo-detected field sweep EPR (EDFS-EPR). The anaerobically reconstituted WhiB4 was EPR silent, suggestive of the presence of an antiferromagnetically coupled [4Fe-4S]^2+^ cluster. Treatment with excess sodium dithionite (DTH) at pH 7.5 caused partial bleaching of the protein's yellowish brown colour (data not shown) and resulted in a fast-relaxing axial EPR spectrum with *g*-tensor components of 2.06 and 1.94 (Fig. 1C). This signal was only observed below 10 K and is consistent with the one-electron reduction of an EPR-silent [4Fe-4S]^2+^ species to a paramagnetic [4Fe-4S]^1+^ cluster with electron spin *S* = ½, and exhibiting the fast spin relaxation typical of Fe-S clusters (Singh *et al*., 2007). A brief description of the spectroscopy methods is included in the supplementary information (SI) (SI note 1). In sum, we demonstrate via anaerobic reconstitution and EDFS-EPR that *Mtb* WhiB4 contains a DTH-reducible 4Fe-4S cluster.

### WhiB4 4Fe-4S cluster is responsive to O_2_ and NO

**Fig. 2:**
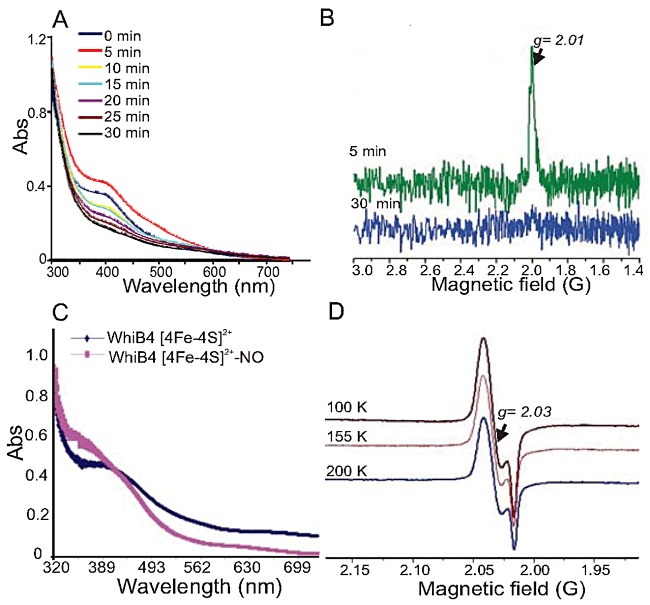
The *Mtb* WhiB4 Fe-S cluster responds to O_2_ and NO. A. UV-visible spectra were obtained before and after exposing anaerobically reconstituted WhiB4 4Fe-4S cluster to air at various time points. Note the time-dependent decrease in the absorbance at 420 nm. B. Air-exposed samples of reconstituted WhiB4 were withdrawn immediately (5 min; green spectrum) or 30 min post exposure (blue spectrum) and analysed by EDFS-EPR. The appearance of a sharp signal at *g* = 2.01 indicates a [3Fe-4S]^1+^ cluster. Conditions for EPR spectroscopy were the same as in Fig. 1C. The influence of NO on WhiB4 was analysed by adding a 10-fold molar excess of proline NONOate (WhiB4:NO) before analysis by UV-visible and cw-EPR spectroscopy. C. UV-visible spectra were acquired before and after addition of proline NONOate. Note the increase in the characteristic monomeric DNIC peak at ∼ 350 nm in the NO-treated sample. D. cw-EPR spectra of NO-treated samples were acquired at a microwave frequency of 9.667 GHz and microwave power of 2 mW at 100, 155 and 200 K. The appearance of a sharp signal around 2.03 and the increase in the intensity of the signal at lower temperatures is consistent with the formation of monomeric DNIC.

Fe-S cluster proteins can function as global regulators by reacting with diatomic gases such as O_2_ and NO (Green and Paget, 2004). To ascertain whether the WhiB4 4Fe-4S cluster is responsive to O_2_, anaerobically reconstituted WhiB4 was exposed to air, and UV-visible spectra were recorded at various time points. After 5 min of exposure, we observed an increase in absorbance at 420 nm, followed by a gradual decline over the next 25 min, as well as a complete loss of protein colour. At ∼ 30 min post air exposure, the absorption spectrum was similar to that of the 2Fe-2S cluster originally present in the freshly purified WhiB4 protein (Fig. 2A). These spectral changes are consistent with an O_2_-induced transformation of a [4Fe-4S] cluster to a [2Fe-2S] cluster (Singh *et al*., 2007). Further incubation of air-exposed WhiB4 resulted in the complete loss of the cluster and the precipitation of the protein (data not shown). Analysis of the absorbance change at 420 nm versus time revealed the loss of ∼ 80% of the 4Fe-4S cluster in 30 min. EDFS-EPR analysis of WhiB4 after brief air exposure (5 min) yielded an unresolved EPR spectrum with *g* = 2.01 (Fig. 2B). The shape and *g*-value of the signal bears strong similarity to a [3Fe-4S]^1+^ cluster, as observed with air-exposed FNR and WhiB3 proteins (Crack *et al*., 2004; Singh *et al*., 2007). This EPR signal was rapidly lost upon prolonged incubation (30 min) of air-exposed WhiB4 (Fig. 2B). Therefore, upon exposure to O_2_, spectral features associated with the 4Fe-4S cluster were rapidly lost in a sequential reaction that yielded apo-WhiB4 via generation of [3Fe-4S]^1+^ and [2Fe-2S] intermediates.

Next, to investigate whether NO can target the WhiB4 4Fe-4S cluster, anaerobically reconstituted WhiB4 was exposed to the fast NO-releasing compound proline NONOate (half-life 1.8 s at pH 7.4) and analysed with UV-visible spectroscopy and continuous wave-EPR (cw-EPR). Exposure to NO leads to the formation of a new chromophoric species with a broad and intense near-UV absorption band at ∼ 350 nm (Fig. 2C). This spectrum is similar to a dinitrosyl-iron dithiol complex (DNIC), wherein the sulphide ligands of the 4Fe-4S cluster are displaced by NO to form [Fe-(NO)_2_] (Cruz-Ramos *et al*., 2002). The EPR spectrum of anaerobically reconstituted, NO-exposed WhiB4 showed a strong EPR signal at *g* = 2.03 (Fig. 2D), suggesting the presence of a monomeric DNIC (Cruz-Ramos *et al*., 2002). This spectrum was visible even at 200 K, indicating that this species was not an Fe-S cluster, whose rapid spin relaxation would preclude observation at such a high temperature. Taken together, these data demonstrate that the WhiB4 Fe-S cluster responds to O_2_ and NO. An important discovery is that the 4Fe-4S cluster of WhiB4 is highly susceptible to O_2_ damage, making it distinct from other WhiB-like proteins, such as WhiB3 and/or WhiB1, whose Fe-S clusters respond very slowly to O_2_ exposure (Singh *et al*., 2007; Smith *et al*., 2010). These data suggest that WhiB4 can function as a sensor of redox signals via its 4Fe-4S cluster.

### WhiB4 regulates growth, redox balance and membrane potential *in vitro*

**Fig. 3:**
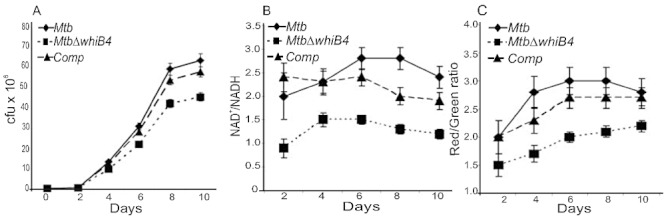
WhiB4 modulates growth, redox homeostasis and membrane potential of *Mtb* under aerobic conditions*.* A. Aerobic growth phenotype of wt *Mtb*, *Mtb*Δ*whiB4* and complemented strains was determined by growing cells in 7H9 medium under aerobic conditions. Growth was monitored at different time intervals by cfu analysis. B. At various days post inoculation, cells were analysed for intracellular redox balance by measuring the poise of NAD^+^/NADH as described in *Experimental procedures*. C. Membrane potential of aerobically growing cells was determined by staining with DiOC_2_. A change in the membrane potential was detected using the average mean fluorescence intensity (Red/Green) emitted by the cells. The fluorescent intensity was normalized to the values obtained upon carbonyl cyanide *m*-chlorophenyl hydrazone (CCCP) treatment. All of the above experiments were carried out at least three times in triplicate and results are given as the mean values and standard deviations.

To understand the role of WhiB4 in the physiology of *Mtb*, we generated a *whiB4* mutant (*Mtb*Δ*whiB4*) in *Mtb* via allelic exchange (Fig. S1A) and confirmed this disruption by PCR (Fig. S1B) and Southern blotting (Fig. S1C). A *whiB4* complemented strain (*comp*.) was also constructed (see *Experimental procedures*). Next, we examined the effect of WhiB4 disruption on the growth of *Mtb* in 7H9 liquid medium under normal aerobic conditions. We observed that *Mtb*Δ*whiB4* cells consistently displayed a slow growth phenotype throughout the life cycle, which was marked by an approximately twofold reduction in colony-forming units (cfu) as compared to wt *Mtb* (Fig. 3A). This result suggests that WhiB4 may play a role in maintaining an optimum overall growth of *Mtb*. We then examined the redox poise of NAD^+^/NADH in *Mtb*Δ*whiB4* during normal culture conditions (see *Experimental procedures*). Because NAD^+^ and NADH are essential for shuttling electrons generated from the oxidation of carbon sources to the respiratory chain, the redox state of NAD^+^/NADH is considered central to redox metabolism (Green and Paget, 2004). As shown in Fig. 3B, we consistently observed an approximately twofold reduced ratio of NAD^+^/NADH in *Mtb*Δ*whiB4* cells as compared to wt *Mtb* and the complemented strain throughout the growth cycle. This result implicates WhiB4 in regulating the redox steady state of the NAD^+^/NADH couple, which contributes to proper growth of *Mtb* under *in vitro* conditions. Since the NAD^+^/NADH redox state should be coupled to the electron transport chain, we analysed the membrane potential (Ψ) of *Mtb*Δ*whiB4* using the cationic fluorescent dye 3,3′-diethyloxacarbocyanine iodide (DiOC_2_) as an indicator of respiration (see *Experimental procedures*). We observed an approximately 1.5-fold reduction in the value of Ψ in *Mtb*Δ*whiB4* cells as compared with wt *Mtb* and the complemented strain (Fig. 3C). In sum, our data implicate WhiB4 in regulating oxidative metabolism and maintaining cellular redox homeostasis during normal aerobic growth of *Mtb*.

### WhiB4 regulates the expression of antioxidant genes in *Mtb*

Next, we performed microarray analysis to examine the function of WhiB4 in *Mtb*. We compared the global transcription profile between wt *Mtb* and *Mtb*Δ*whiB4* grown in 7H9 culture medium. Microarray data revealed that expression of only 26 genes was altered in *Mtb*Δ*whiB4* under normal growing conditions. Of these 26 differentially regulated genes, expression of 25 was upregulated by a factor of 1.3–2 in *Mtb*Δ*whiB4*. To identify differentially expressed genes in *Mtb*Δ*whiB4* that were of statistical significance, we performed SAM analysis (Significance Analysis of Microarray) as described in *Experimental procedures*. The SAM results confirmed that 23 genes (Table S1) were indeed regulated by WhiB4 at the most stringent levels of analysis (false discovery rate = 0). Furthermore, we confirmed our microarray data by analysing the expression of several WhiB4-regulated genes via quantitative real-time PCR (qRT-PCR). The expression of these genes in wt *Mtb*, *Mtb*Δ*whiB4* and *whiB4* complemented strain was normalized against 16S rRNA expression levels (Table S2).

Consistent with the role of WhiB4 in sensing intracellular redox stress, expression data revealed that several genes associated with antioxidant systems were upregulated in *Mtb*Δ*whiB4*. Conventional antioxidant genes such as *ahpC* and *ahpD* encoding alkyl hydroperoxide reductase (Chen *et al*., 1998) were induced in the mutant. A new discovery was the upregulation of unconventional antioxidant systems in *Mtb*Δ*whiB4*. For example, an operon-like cluster of four genes (Rv3249c–Rv3252c) encoding components of the rubredoxin system (*rubA*, *rubB* and *alkB*) was also upregulated in *Mtb*Δ*whiB4*. It has been shown that the rubredoxin system in bacteria (e.g. *Desulfovibrio vulgaris*) prevents auto-oxidation of redox enzymes and reduces intracellular levels of H_2_O_2_ and superoxide (Coulter and Kurtz, 2001). Another important inducible set of genes (Rv0692–Rv0694) shows similarity to enzyme complexes involved in the biosynthesis of the novel redox cofactor pyrroloquinoline quinone (PQQ) (Haft, 2011). PQQ is a powerful antioxidant that protects proteins and DNA from oxidative damage by scavenging ROI (Misra *et al*., 2004). Microarray data revealed that the expression of *whiB4* was also induced in *Mtb*Δ*whiB4*, suggesting an autorepressor function of WhiB4. Presence of the initial 96 bases of the *whiB4* gene in *Mtb*Δ*whiB4* likely facilitated the identification of the abortive *whiB4* transcript in these microarray experiments.

We observed that *whiB6* was also induced in *Mtb*Δ*whiB4*. A recent comparison of all WhiB members demonstrates that *whiB6* expression exhibits the highest degree of stress-responsiveness (Geiman *et al*., 2006). The upregulation of *whiB6* in the absence of WhiB4 suggests that WhiB6 could be a compensatory mechanism for *Mtb* to sense and respond to intracellular redox stress. Finally, we show that several members of the PE-PPE family (PE35, PPE68 and PPE19) were also differentially regulated in *Mtb*Δ*whiB4* (Table S1).

It is also noteworthy that there was a significant overlap between the genes regulated in *Mtb*Δ*whiB4* and those differentially expressed in *Mtb* upon exposure to the anti-tuberculosis drug Isoniazid (INH). These include the components of the FASII operon involved in mycolic acid biosynthesis (Rv2243-Rv2246), the *iniBAC* operon, *ahpC-ahpD*, *nrdB*, Rv3250c–Rv3252c and *whiB4* (Betts *et al*., 2003; Karakousis *et al*., 2008). Peroxidative activation of INH by KatG is known to induce intracellular redox stress by generating endogenous ROI and RNI (Timmins and Deretic, 2006). Thus induction of INH-responsive genes in *Mtb*Δ*whiB4* further implicates WhiB4 in sensing and maintaining endogenous redox balance. Taken together, our results suggest that WhiB4 regulates a specific set of genes involved in protecting *Mtb* from environmental stresses encountered during infection.

### WhiB4 is a redox-dependent DNA-binding protein

Having established that WhiB4 influences the expression of stress responsive genes in *Mtb*, we now sought to determine whether WhiB4 regulates gene expression by directly binding to DNA. To show this, we examined the interaction of WhiB4 with the promoter regions of *ahpC* and *whiB4*. We reconstituted the [4Fe-4S]^2+^ form of WhiB4 (holo-WhiB4) under anaerobic conditions, which was confirmed via UV-visible spectroscopy as previously described. Reduced ([4Fe-4S]^1+^) and oxidized ([4Fe-4S]^2+^) holo-WhiB4 were assayed for DNA-binding activity under anaerobic conditions. We did not observe any noticeable DNA-binding activity of either reduced or oxidized holo-WhiB4 to either *ahpC* (Fig. S2) or *whiB4* promoter regions (data not shown).

**Fig. 4:**
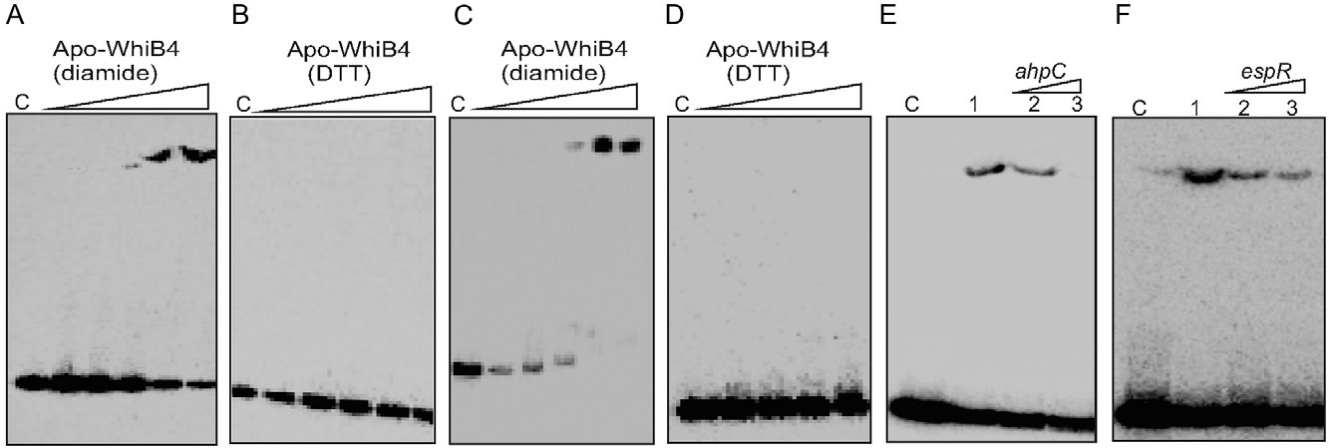
DNA binding activity of WhiB4. A–D. Apo-WhiB4 was prepared as described in *Experimental procedures*. The concentrations of apo-WhiB4 used for EMSAs were 0.1, 0.2, 0.4, 0.8 and 1 µM. EMSA reactions were performed with 0.2 nM γ-^32^P-labelled *ahpC* (A and B) and *whiB4* (C and D) promoter DNA fragments. DNA binding of apo-WhiB4 in the presence of thiol-oxidant, diamide (A and C) or thiol-reductant, DTT (B and D). C: DNA binding in the absence of WhiB4 in each panel. E and F. Sequence preference of WhiB4 for DNA binding. EMSAs were performed using γ-^32^P-labelled *ahpC* promoter DNA with 800 nM of apo-WhiB4 in the presence of 50 mM diamide. The DNA binding was competed using increasing concentrations of unlabelled *ahpC* (specific) or *espR* (non-specific) promoter DNA. Lane 1 in (E) and (F): WhiB4:*ahpC* promoter complex. WhiB4 DNA binding was competed using 10-fold (lane 2) and 50-fold (lane 3) molar excess of either unlabelled *ahpC* (E) or *espR* (F) promoter DNA. C: DNA binding in the absence of WhiB4 in each panel.

Since WhiB4 rapidly loses its 4Fe-4S cluster upon air oxidation to generate apo-WhiB4, we determined whether degradation of the Fe-S cluster activated DNA binding of WhiB4. Apo-WhiB4 contains four Cys residues that have been shown to undergo thiol-oxidation or reduction in response to diamide or DTT respectively (Alam *et al*., 2009). In the presence of diamide, apo-WhiB4 binds to the promoter regions of *ahpC* and *whiB4* in a concentration-dependent manner (Fig. 4A and C). In contrast, DNA binding by apo-WhiB4 was completely lost in the presence of DTT (Fig. 4B and D). Lastly, we independently replaced the four Cys residues in WhiB4 with alanine and analysed the DNA binding capacity of mutant proteins using *ahpC* promoter. The absence of DNA binding in the case of Cys mutants suggests that the oxidation of four Cys residues is essential for the DNA binding activity of apo-WhiB4 (Fig. S3). These results demonstrate that DNA binding of apo-WhiB4 is regulated by the redox state of the Cys residues and suggest that O_2_-activated Fe-S cluster degradation and subsequent oxidation of Cys thiols serve as a switch to activate WhiB4 for its role in gene regulation.

### WhiB4 binds DNA in a sequence-independent manner

We next tested whether apo-WhiB4 binds DNA specifically or non-specifically by competition assays. Since *ahpC* was identified as one of the genes regulated by WhiB4, we used its promoter DNA fragment to examine whether binding was specific, while a non-related promoter fragment of a *Mtb* gene (Rv3849, *espR*) was utilized as a negative control. We found that a 50-fold molar excess of the unlabelled *ahpC* promoter fragment completely prevented binding of oxidized apo-WhiB4 to the labelled *ahpC* promoter (Fig. 4E). However, the same concentrations of an unlabelled *espR* promoter fragment also reduced apo-WhiB4 binding to approximately similar extent, suggesting non-specific DNA binding activity of apo-WhiB4 (Fig. 4F). The sequence-independent DNA binding of the oxidized apo-WhiB4 was further analysed by examining its interaction with the unrelated promoter fragments derived from *Mtb* genes *pks3* (Rv1180) and *rrnA*. As shown in Fig. S4, apo-WhiB4 displayed efficient binding to both the promoters.

**Fig. 5:**
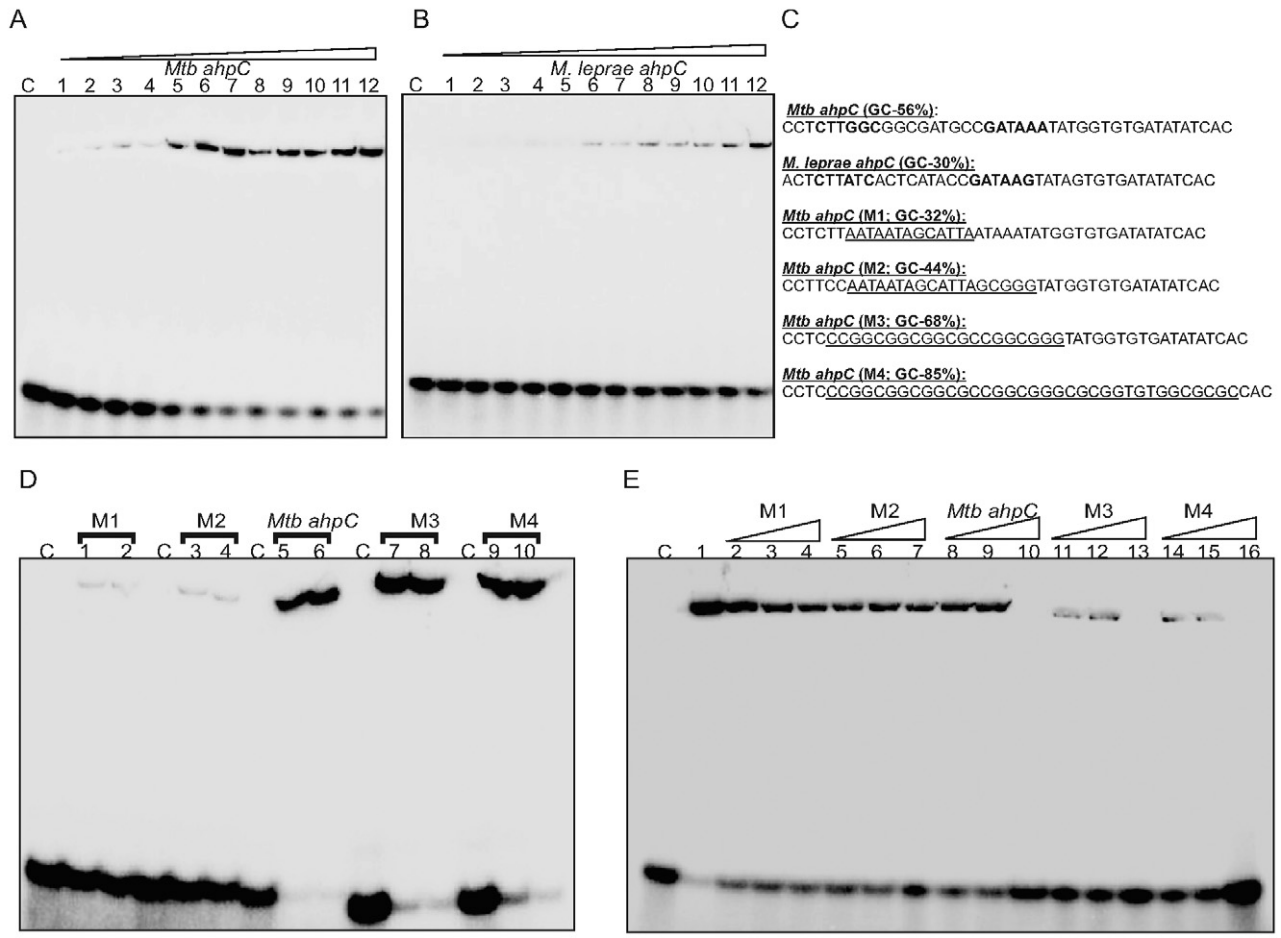
WhiB4 binds to GC-rich DNA. A and B. Concentration-dependent binding of oxidized apo-WhiB4 to a 40 bp γ-^32^P-labelled DNA fragment derived from (A) *Mtb* and (B) *M. leprae ahpC* promoter regions containing the OxyR-binding motif. The concentrations of oxidized apo-WhiB4 were 0.25, 0.50, 0.75, 1.00, 1.25, 1.50, 1.75, 2.0, 2.25, 2.50, 2.75, 3.00 µM. The *K*_d_ for both the fragments was calculated by measuring the intensity of free and protein bound DNA using ImageJ software. Note that apo-WhiB4 binds with higher affinity to the GC-rich *Mtb* OxyR recognition sequence as compared with the AT-rich *M. leprae* OxyR recognition sequence within the *ahpC* promoter. C. DNA fragments containing mutations to modify the GC content of the *ahpC* promoter region (OxyR binding core motif is shown in bold). The mutated sequences in various DNA fragments (M1–M4) are underlined. These fragments were subjected to EMSA analysis. D. EMSAs were carried out using 0.8 and 1 µM oxidized apo-WhiB4 and 0.2 nM γ-^32^P-labelled DNA fragments. Note that apo-WhiB4 showed enhanced binding to M3 (68% GC) and M4 (85% GC) as compared with M1 (32% GC) and M2 (44% GC) fragments. E. Competition assay using high and low GC DNA fragments. Lane 1: oxidized apo-WhiB4:*ahpC* promoter complex. DNA binding was competed using 10-fold (lanes 2, 5, 8, 11 and 14), 20-fold (lanes 3, 6, 9, 12 and 15) and 50-fold (lanes 4, 7, 10, 13 and 16) molar excess of unlabelled DNA fragments as indicated in the figure. C: DNA binding in the absence of WhiB4 in each panel.

To gain the mechanistic understanding of WhiB4 non-specific DNA binding activity, we further extended our study on the regulation of *ahpC* expression by WhiB4. The expression of *ahpC* is controlled via OxyR in other mycobacterial species such as *M. leprae* and *M. avium*. In *M. leprae*, OxyR binds to its consensus sequence in the *ahpC* promoter (Pagan-Ramos *et al*., 1998), whereas in *Mtb*, both the *oxyR* ORF and its consensus sequence in the *ahpC* promoter region contain multiple mutations (Pagan-Ramos *et al*., 1998). We addressed whether WhiB4 could discriminate between the OxyR-binding motif present in the promoter sequences of *ahpC* from *Mtb* and *M. leprae*. To do this, we performed DNA binding assays using an approximately 40 bp oligonucleotide containing *Mtb* or *M. leprae* OxyR binding sequence. Our data demonstrated that apo-WhiB4 binds to the fragment containing the *Mtb* OxyR-binding motif in a concentration-dependent manner with a dissociation constant (*K*_d_) of approximately 1 µM (Fig. 5A). In contrast, we found that apo-WhiB4 binding to the *M. leprae* fragment was significantly reduced (∼ 10% of the *M. leprae* as compared with ∼ 60% of the *Mtb ahpC* promoter fragment was bound at 1 µM of apo-WhiB4) (Fig. 5B). These results indicate that WhiB4 prefers OxyR binding sequence in the promoter of *Mtb ahpC* over *M. leprae ahpC*.

### *Mtb* WhiB4 preferentially binds to GC-rich sequences

Since WhiB4 binds to the *Mtb ahpC* promoter region, we used this region to search for consensus sequences in the promoter regions of other genes regulated by WhiB4. We found no consensus patterns upon alignment of the sequences ∼ 400 bp upstream of the start codon for the genes regulated by WhiB4. However, closer inspection of the OxyR consensus sequence in the *ahpC* promoter region revealed that it is relatively GC-rich (56%) in *Mtb* as compared to *M. leprae* (30%). The upstream sequences (∼ 400 bp) from the other 23 WhiB4 regulated genes showed that they were also GC-rich (56–70%). This indicates that WhiB4 could bind DNA non-specifically with a preference for GC-rich sequences.

To examine this possibility, we randomly modified the GC content of the OxyR-binding motif in the *Mtb ahpC* promoter. Figure 5C lists the mutations tested in this experiment. First, we confirmed that apo-WhiB4 binding to the 40 bp *Mtb ahpC* promoter fragment can be efficiently outcompeted by the non-specific promoter regions of *Mtb espR* (58% GC) and *cfp10* (60% GC) (Fig. S5). Next, DNA binding was performed using randomly modified fragments of varying GC content and oxidized apo-WhiB4. Figure 5D shows that apo-WhiB4 binds with the wt *Mtb ahpC* promoter (56% GC). However, DNA binding was significantly reduced in the case of mutant promoters M1 and M2 with a GC content of 32 and 44% respectively (Fig. 5D). Furthermore, DNA binding was fully restored when the GC content was increased to 68 and 85% in the M3 and M4 fragments respectively (Fig. 5D). Kinetic analysis confirmed that apo-WhiB4 binds to the M3 and M4 fragments with *K*_d_ values of 0.6 and 0.4 µM respectively (Fig. S6). These findings were separately verified by competition assays using wt *ahpC*, M1, M2, M3 and M4 fragments. Figure 5E demonstrates that competition with a 50-fold molar excess of specific wt *ahpC* promoter (56% GC) resulted in complete loss of DNA binding, whereas the same concentration of either M1 (32% GC) or M2 (44% GC) resulted in a modest loss of WhiB4 DNA binding. In contrast, we observed that only a 10-fold molar excess of M3 (68% GC) and M4 (85% GC) fragments was required to completely abolish WhiB4 DNA binding (Fig. 5E).

Lastly, we found that WhiB4 DNA binding to the wt *ahpC* promoter was efficiently competed by excessive poly (dG:dC) and poly (dI:dC) but not by poly (dA:dT) (Fig. S7). In sum, data generated suggest that WhiB4 is a non-specific DNA-binding protein with a preference for GC-rich DNA sequences.

### DNA minor groove binding drugs compete with WhiB4 for DNA binding

Since AT- and GC-rich sequences are known to influence groove topology in DNA (Rohs *et al*., 2009), we evaluated the ability of major and minor groove binding drugs to compete against WhiB4:DNA interactions. We used actinomycin D and chromomycin A3, as minor groove binding drugs, and methyl green, a major groove binding drug in our competition assays. As shown in Fig. S7, actinomycin D and chromomycin A3 effectively abolished binding of WhiB4 to the *ahpC* promoter fragment, whereas methyl green had no effect. These results suggest that WhiB4 binds to GC-rich DNA through the minor groove. The ability of minor groove-binding proteins to regulate expression by remodelling DNA architecture (Bewley *et al*., 1998) suggests that WhiB4 could repress expression of stress genes by binding in the minor groove of GC-rich promoters and changing the conformation of the promoter regions.

### Apo-WhiB4 represses transcription *in vitro*

**Fig. 6:**
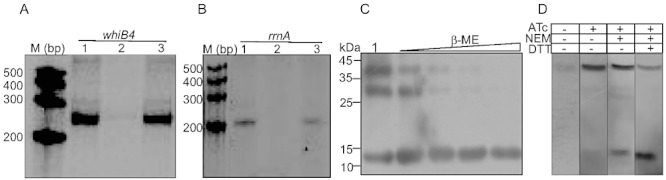
A and B. Effect of WhiB4 on *in vitro* transcription. Single round transcription assays show that RNAP-σ^A^ holoenzyme was proficient in directing transcription from *whiB4* (A, lane 1) and *rrnA* (B, lane 1) promoters. 50 nM of *whiB4* and *rrnA* promoter DNA fragments were pre-incubated with either 2 µM oxidized apo-WhiB4 (A, lane 2; B, lane 2) or reduced apo-WhiB4 (A, lane 3; B, lane 3) and subjected to transcription by RNAP-σ^A^. M: RNA marker (Century™ Marker Template, Ambion). C. Disulphide bond formation induces oligomerization of apo-WhiB4 *in vitro*. Five micrograms of apo-WhiB4 is either oxidized by atmospheric O_2_ (lane 1) or reduced by 50 mM, 100 mM, 200 mM and 400 mM β-ME and resolved on a 12% non-reducing SDS-PAGE. Apo-WhiB4 bands were visualized by Western blot analysis using anti-His antibody. The ∼ 14 kDa, ∼ 28 kDa and ∼ 42 kDa bands correspond to the His-tagged apo-WhiB4-monomer, -dimer and -trimer. D. *In vivo* existence of disulphide-linked oligomers of apo-WhiB4. Aerobically grown *Msm* WhiB4 FLAG-tag strain was either uninduced or induced with ATc and 30 µg of cell-free extract was analysed by non-reducing Western blot using anti-FLAG antibody. Note that a significant portion of the FLAG-tagged apo-WhiB4 exists as a trimer in ATc induced *Msm* cells. To minimize the possibility of O_2_-induced thiol oxidation and subsequent oligomerization of apo-WhiB4 during cell-free extract preparation, *Msm* cells expressing FLAG-tagged WhiB4 were pretreated with the thiol-alkylating agent NEM. Note the presence of apo-WhiB4 trimer in the NEM-pretreated sample. A significant loss of apo-WhiB4 oligomerization upon DTT reduction further suggests the presence of intermolecular disulphide-linked oligomers *in vivo*.

To understand the influence of apo-WhiB4 DNA binding on transcription, we performed *in vitro* transcription assays using a highly sensitive *Mycobacterium smegmatis* (*Msm*) RNA polymerase holoenzyme containing stoichiometric concentrations of the principal sigma factor, SigA (RNAP-σ^A^) (see SI note 2). The *Msm* RNAP-σ^A^ specifically binds to and activates transcription from the σ^A^-dependent promoters (China and Nagaraja, 2010). To identify which of the WhiB4-regulated genes are recognized and transcribed by RNAP-σ^A^, we performed single round transcription assays using the promoter regions of *ahpC*, *iniB* and *whiB4*. The *rrnA* promoter was used as a positive control for σ^A^ -specific transcription. In these assays, RNAP-σ^A^ transcription activity was not detected at the *ahpC* or *iniB* promoters (data not shown). However, RNAP-σ^A^ catalyses transcription specifically from *whiB4* and *rrnA* promoters, producing a single major transcript of ∼ 260 bp and ∼ 210 bp respectively (Fig. 6A and B). The size of *rrnA* transcript is in agreement with the reported transcriptional start point (TSP) for the *rrnA* ORF (Verma *et al*., 1994; Gonzalez-y-Merchand *et al*., 1996), confirming the specificity of RNAP-σ^A^ activity (see SI note 3). *In silico* promoter analysis of *whiB4* using Artificial Neural Networks Promoter Prediction tool (ANNPP 2.2, http://www.fruitfly.org/seq_tools/promoter.html) program (Kalate *et al*., 2003) revealed the presence of a putative −10 like sequence and a potential TSP, which corresponds to the size of the *whiB4* transcript detected in our assays (see SI note 3). Next, we assessed the influence of apo-WhiB4 on the transcription from *whiB4* and *rrnA* promoters. Addition of oxidized apo-WhiB4 completely inhibited transcription from the *whiB4* and *rrnA* promoters, whereas reduced apo-WhiB4 restored the transcription to normal levels (Fig. 6A and B). These data demonstrate that WhiB4 affects transcription in a redox-dependent, but promoter-sequence-independent manner. The non-specific DNA binding and transcriptional inhibition exhibited by WhiB4 is mechanistically similar to several nucleoid-associated proteins (NAPs) in bacteria (Colangeli *et al*., 2007; Dillon and Dorman, 2010).

### Apo-WhiB4 oligomerizes *in vitro* and *in vivo*

To examine the oligomeric status of oxidized and reduced apo-WhiB4, we performed non-reducing Western blot analysis of apo-WhiB4 using anti-His antibody. Western blot analysis of air-oxidized apo-WhiB4 yielded three bands of ∼ 14 kDa, ∼ 28 kDa and ∼ 42 kDa that correspond to the size of a full-length His-tagged apo-WhiB4 monomer, dimer and trimer respectively (Fig. 6C). In contrast, the oligomeric apo-WhiB4 forms were gradually reduced to monomeric form by increasing amounts of β-mercaptoethanol (β-ME) (Fig. 6C). The presence of SDS-resistant oligomers under non-reducing conditions indicates the formation of intermolecular disulphide bonds between the Cys thiols of apo-WhiB4.

To determine whether the formation of disulphide bonded oligomers might be of physiological significance, we used an anhydrotetracycline (ATc)-inducible *E. coli*-mycobacterial shuttle plasmid pEXCF-*whiB4* containing FLAG-tagged *whiB4*. The pEXCF-*whiB4* was electroporated in *Msm* and WhiB4 was conditionally expressed using ATc as described in *Experimental procedures*. The presence of WhiB4 was detected in the cell-free extract of *Msm* by non-reducing Western blot analysis using anti-FLAG antibody. In the absence of ATc, we detected a faint band of apo-WhiB4 migrated as a trimer, indicating the presence of disulphide-linked oligomers (Fig. 6D). Furthermore, overexpression of WhiB4 by ATc demonstrates that apo-WhiB4 mainly exists as a trimer (Fig. 6D). To conclusively determine that disulphide-linked oligomers existed in the intact cells and were not generated during cell extract preparation, we pretreated *Msm* expressing FLAG-tagged WhiB4 with the membrane-permeable alkylating agent, *N*-ethylmaleimide (NEM) as described in *Experimental procedures*. NEM alkylates free-SH groups, blocking their participation in disulphide bond formation, but does not disrupt the existing disulphide bonds. As shown in Fig. 6D, a significant portion (∼ 90%) of apo-WhiB4 exists as a trimer, whereas only a fraction of apo-WhiB4 is present in a reduced monomer form under alkylating conditions in the intact cells. Furthermore, DTT reduction of the NEM-treated samples converted a major fraction of the oligomeric apo-WhiB4 to the monomeric form (Fig. 6D). These data show that apo-WhiB4 oligomerizes via intermolecular disulphide bonds inside aerobically growing *Msm*, suggest that some trimeric apo-WhiB4 could be disulphide linked in *Mtb* cells to bind DNA and repress transcription.

### WhiB4 regulates the survival of *Mtb* upon oxidative stress

Having shown that WhiB4 modulates the expression of genes involved in dissipating oxidative stress, we analysed the sensitivity of *Mtb*Δ*whiB4* to ROI generators such as CHP and menadione. We found that *Mtb*Δ*whiB4* survived 20-fold better than wt *Mtb* upon exposure to 25 and 50 µM CHP (Fig. S8A). Consistent with this, *Mtb*Δ*whiB4* also displayed 10-fold better survival against 40–50 µM menadione compared with wt *Mtb* (Fig. S8B). This resistance phenotype was reversed in the *whiB4* complemented strain (Fig. S8A and B). These observations suggest that the increased resistance of *Mtb*Δ*whiB4* to oxidative stress could be due to the induction of antioxidant systems in the mutant.

### WhiB4 modulates the induction of antioxidant genes upon oxidative stress

Here we examine whether WhiB4 regulates the expression of antioxidants in response to oxidative stress. We exposed wt *Mtb* and *Mtb*Δ*whiB4* to CHP and analysed the expression by qRT-PCR. Exposure to CHP resulted in the induction of *ahpC*, *ahpD*, *rubA*, *pqqE*, Rv3249c and *whiB6* in wt *Mtb* as compared with unstressed cells (Table S3). However, the expression of these genes was consistently higher in the CHP-treated *Mtb*Δ*whiB4* as compared with wt *Mtb* (Table S3). Furthermore, we detected a significant downregulation of *whiB4* in the CHP-treated wt *Mtb*, whereas the expression of partial *whiB4* transcript was moderately induced in the CHP-treated *Mtb*Δ*whiB4* (Table S3). The effect of CHP on the expression was restored to near wt *Mtb* levels in the *whiB4* complemented strain. In sum, our data suggest that WhiB4 modulates the ability of *Mtb* to resist oxidative stress by the controlled activation of antioxidant response.

### WhiB4 modulates survival of *Mtb* during infection of macrophages

Given that WhiB4 regulates expression of antioxidant genes, resulting in differential survival of *Mtb* in response to oxidative stress *in vitro*, and that *Mtb* encounters oxidative stress during infection, we hypothesize that WhiB4 protein plays an important role in the infection process. To test this hypothesis, we performed a series of experiments with cultured cells and whole animal systems, comparing the performance of *Mtb*Δ*whiB4* to wt *Mtb.*

**Fig. 7:**
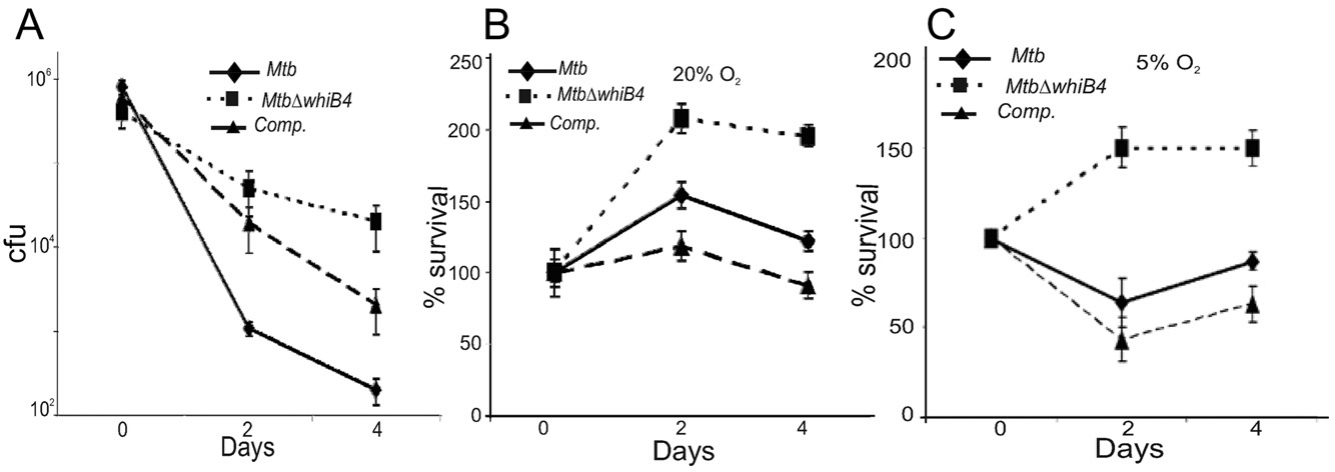
WhiB4 regulates survival of *Mtb* inside macrophages. A. rIFNγ and LPS-activated Raw264.7 cells were infected with *Mtb* strains at a moi of 10 and growth was monitored over time by cfu. B and C. To investigate the role of O_2_ tension, phorbol 12-myristate 13-acetate (PMA)-stimulated THP-1 human monocytic cell lines were maintained at 20% O_2_ (B) and 5% O_2_ (C) and infected by various *Mtb* strains at a moi of 10. For each strain, the cfu at each time point are expressed relative to the cfu at time 0. In each case, data shown are the average of three experiments performed in triplicate; the error bars indicate standard deviation in each group.

Infection of macrophages exposes *Mtb* to oxidative and nitrosative stresses (Gordon and Read, 2002). Therefore, we first examined the phenotype of *Mtb*Δ*whiB4* in resting Raw264.7 macrophages. *Mtb*Δ*whiB4* grew at a level similar to wt *Mtb* inside resting macrophages (Fig. S9). Since macrophages activated by IFN-γ and LPS generate a more hostile environment by inducing vacuole acidification, nutrient depletion, and enhanced ROI/RNI production to constrain bacterial growth (James *et al*., 1995; Schaible *et al*., 1998), we analysed the influence of the activation status of the infected macrophages on the survival of *Mtb*Δ*whiB4*. In activated macrophages, we observed an approximately 50-fold enhanced survival of *Mtb*Δ*whiB4* as compared with wt *Mtb* at day 2 post infection (Fig. 7A). This difference was further amplified to approximately 1000-fold at day 4 post infection (Fig. 7A). The original survival phenotype of *Mtb*Δ*whiB4* was significantly restored in the complemented strain. This trend was confirmed in three independent experiments.

Until now, we have shown the effect of WhiB4 in controlling *Mtb* survival inside murine macrophages cultivated at ambient O_2_ pressure (20%; pO_2_ 140 mmHg). However, 20% O_2_ is not physiologically relevant because human tissues maintain O_2_ levels of 5% (36 mmHg) (Meylan *et al*., 1992a, b). Interestingly, it has been shown that human monocytes displayed higher ROI production and effectively controlled the replication of *Mtb* when cultured at 5% O_2_ (Meylan *et al*., 1992b). To investigate the role of WhiB4 in settings closer to physiological conditions, we examined the survival of *Mtb*Δ*whiB4* in THP-1 human monocytic cell lines cultivated at 20% (ambient) and 5% (reduced) O_2_ levels. As shown in Fig. 7B and C, wt *Mtb* grew normally at higher O_2_ tension (20%), whereas lower pO_2_ consistently reduced its growth at early time points, followed by a slow increase in the survival. This intracellular growth pattern of wt *Mtb* at 5% O_2_ was in agreement with the original study reported by Meylan *et al*. (1992b). In contrast, *Mtb*Δ*whiB4* grew more robustly than wt *Mtb* inside THP-1 macrophages at both 20% and 5% O_2_ (Fig. 7B and C), at all time points of infection, with the most striking difference at low O_2_ tension (5%) (Fig. 7C). Lastly, at both O_2_ concentrations, the growth phenotype of the complemented strain was restored to wild-type levels (Fig. 7B and C). This phenotype of *Mtb*Δ*whiB4* was confirmed in three independent experiments. Taken together, the data generated from *in vitro* and macrophage experiments clearly suggest that WhiB4 functions as a sensor of oxidative stress and modulates the survival of *Mtb* in response to enhanced antimycobacterial activity in macrophages.

### WhiB4 regulates *Mtb* persistence and dissemination *in vivo*

**Fig. 8:**
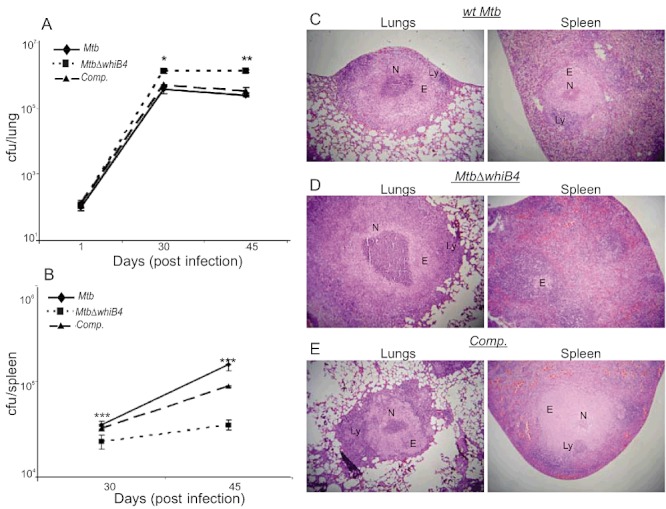
WhiB4 modulates *in vivo* survival and pathology of *Mtb* in guinea pigs. Outbred Hartley guinea pigs (*n* = 5) given an aerosol challenge of *Mtb* were assessed for bacterial burden in lungs (A) and spleen (B), and for the severity of lung and spleen pathology (C–E). Statistical significance for the pulmonic and splenic bacterial load was obtained by comparing wt *Mtb* and *Mtb*Δ*whiB4* strains: *P < 0.05, **P < 0.01, ***P < 0.001. Haematoxylin and eosin stained lung and spleen sections (30 days post infection) from guinea pigs infected with wt *Mtb* (C), *Mtb*Δ*whiB4* (D) and the *whiB4* complemented (Comp.) strains (E). The pathology sections show granulomas containing areas of necrosis (N), epithelioid cells (E) and lymphocytes (L). All images were taken at 4× magnification. Error bars represent the standard error of the mean.

Till date, there has been only one study showing the importance of a WhiB protein (WhiB3) in regulating the pathogenesis of *Mtb* in animal models (Steyn *et al*., 2002). The observation that WhiB4 modulates oxidative stress survival prompted us to investigate the effect of this protein during infection in guinea pigs. Aerosol infection of outbred Hartley guinea pigs showed a clear growth benefit of *Mtb*Δ*whiB4* as compared with wt *Mtb* in the lungs of animals. Results show that nearly identical numbers of bacteria were seeded in the lungs of animals infected with various strains at day 1 post infection (Fig. 8A). At day 30 post infection, the number of bacteria present in the lungs of *Mtb*Δ*whiB4* infected guinea pigs was approximately sixfold higher (*P* < 0.001) than those infected with wt *Mtb* (Fig. 8A). Importantly, guinea pigs were able to control wt *Mtb* growth following activation of the adaptive immune response (∼ 45 days post infection). At this time, the bacillary load of *Mtb*Δ*whiB4* was approximately eightfold higher than wt *Mtb* (Fig. 8A). Interestingly, and in contrast to our lung data, bacterial number in the spleen at 30 and 45 days post infection was lesser for *Mtb*Δ*whiB4* than the wt *Mtb* (*P* < 0.001), suggesting that WhiB4 is necessary for dissemination and/or colonization of *Mtb* to the spleen (Fig. 8B). Finally, the *in vivo* phenotype of *Mtb*Δ*whiB4* was abolished in case of animals infected with the *whiB4* complemented strain (Fig. 8A and B).

### *Mtb* WhiB4 regulates pathogenesis in guinea pigs

To further investigate the unique phenotype exhibited by *Mtb*Δ*whiB4 in vivo*, we performed a histopathological analysis of parts of the lungs and spleen from infected animals. We did not observe a significant difference in the number of granulomas present in the lung lesions of animals infected with either wt *Mtb* or *Mtb*Δ*whiB4*. However, the pulmonic lesions of *Mtb*Δ*whiB4* infected animals showed larger and more necrotic granulomas as compared with animals infected with wt *Mtb* or complemented strain at 30 and 45 days post infection (Figs 8C–E and S10), suggesting severe pathological changes induced by the mutant.

The spleen of wt *Mtb* infected guinea pigs displayed larger granulomas with well delineated central necrotic areas at 30 days post infection (Fig. 8C). In contrast, the splenic parenchyma of *Mtb*Δ*whiB4* infected animals displayed no evidence of organized granulomas (Fig. 8D). At 45 days post infection, the splenic lesions in *Mtb*Δ*whiB4*-infected animals showed the presence of granulomas with modest necrosis; however, the pathology remained less severe as compared with wt *Mtb* infected spleen (Fig. S10). These data suggest that the high load of *Mtb*Δ*whiB4* induces severe lung pathology, whereas its delayed dissemination results in slow progression of spleen pathology.

Taken together, these results reveal a previously unidentified phenotype of *Mtb in vivo* and suggest that WhiB4 is important for regulating survival in the lungs and dissemination of pathogen to the extrapulmonary tissues.

## Discussion

In the present work, we provide a new mechanistic insight into the molecular function of WhiB4 in *Mtb* and demonstrate that WhiB4 binds DNA and regulates the expression of antioxidant and stress responsive genes in *Mtb*. Our data suggest that WhiB4 is a part of a system that carefully controls the expression of antioxidant genes in *Mtb* to modulate survival and dissemination *in vivo*.

*Mtb* WhiB4 contains a redox responsive [4Fe-4S]^2+^ cluster, which degrades within minutes of exposure to O_2_. The presence of extremely O_2_-labile 4Fe-4S cluster was not reported in other WhiB family members (Jakimowicz *et al*., 2005; Singh *et al*., 2007; Smith *et al*., 2010), suggesting that differences in the redox potential of Fe-S clusters may control the function of WhiB proteins. As is the case for FNR and SoxR (Green and Paget, 2004), NO also targets the WhiB4 Fe-S cluster, suggesting that WhiB4 activity can be modulated by nitrosylation of its Fe-S cluster during infection. Our results are in agreement with an earlier report showing the presence of a 4Fe-4S cluster in WhiB4 (Alam *et al*., 2007). However, the redox state and the O_2_/NO reaction intermediates of the WhiB4 4Fe-4S cluster remained uncharacterized. Our study provides biochemical characterization of redox, O_2_ and NO sensing properties of the Fe-S cluster in WhiB4.

Biochemical and expression data suggest that WhiB4 maintains redox homeostasis by regulating the expression of oxidative stress response systems in *Mtb*. We found that almost all of the WhiB4 repressed genes were differentially expressed under stress condition(s) that mimic the *in vivo* environment. For example, *ahpC-ahpD* is activated in response to ROI, RNI, and inside activated macrophages (Schnappinger *et al*., 2003). Similarly, *rubA*, *rubB and alkB* were found to be differentially regulated under acidic pH, hypoxic conditions, starvation, and inside macrophages (Betts *et al*., 2002; Schnappinger *et al*., 2003; Kim *et al*., 2008). Expression of *icl, nrdB, iniB, kasA, kasB, acpM, fabD, whiB4* and *whib6* is regulated by a wide variety of stress conditions such as acidic pH, drug treatment (INH/ETH), oxidative stress, detergent (SDS) and heat stress (Fisher *et al*., 2002; Betts *et al*., 2003; Schnappinger *et al*., 2003; Geiman *et al*., 2006).

To explore the underlying mechanism of WhiB4 function, we characterized its redox and DNA binding properties and found that the loss of Fe-S cluster and the subsequent oxidation of the co-ordinating Cys thiols stimulate WhiB4 DNA binding and transcriptional repression. Furthermore, oxidation of Cys thiols stimulates intermolecular disulphide bond formation between apo-WhiB4 monomer in a DTT-reversible manner. In addition, we detected the presence of disulphide-linked endogenous oligomers of apo-WhiB4 in *Msm* overexpressing FLAG-tagged WhiB4. Taken together, these findings suggest that the formation of stabilized oligomeric apo-WhiB4 by one or more disulphide bonds may be the regulatory mechanism that controls WhiB4 DNA binding and transcriptional activity.

Our result indicating the presence of disulphide-linked apo-WhiB4 inside the reduced environment of mycobacterial cytosol is not unprecedented. In *Rhodobacter capsulatus*, CrtJ (a DNA binding repressor) and RegB (a sensor kinase) contain redox-active Cys residues that exist in a disulphide bonded form during aerobic culturing conditions (Masuda *et al*., 2002; Swem *et al*., 2003). Moreover, Cys residues flanked by basic amino acids are known to have a significantly lower *pKa*, leading to deprotonation of their sulphydryl group at physiological pH. Interestingly, WhiB4 contains several cationic amino acids that surround the Cys residues (Lys36–Cys37–Arg38, Cys59–Arg60), which may decrease Cys residues *pKa*, resulting in the formation of Cys thiolates and disulphide bonds in the cytosol of mycobacteria.

Although, metal-based sensors (e.g. SoxR, IscR, FNR) require redox-active Fe-S cluster for DNA binding and transcription (Green and Paget, 2004), recent studies involving *E. coli* IscR, *B. cereus* FNR, *Mtb* WhiB3 and *Mtb* WhiB1 have identified a mechanism analogous to WhiB4, in which the loss of Fe-S and redox modifications of Cys thiols activate their role in gene regulation (Esbelin *et al*., 2008; Nesbit *et al*., 2009; Singh *et al*., 2009; Smith *et al*., 2010). While the *in vitro* data suggest that disulphide bond formation may be responsible for the redox-sensing and DNA binding activity of WhiB4, it cannot be discounted that other Fe-S cluster intermediates (e.g. 3Fe-4S, 2Fe-2S) or oxidized Cys derivatives (e.g. sulphenic acid) may be occurring inside *Mtb* cells that affect the activity of WhiB4.

The specificity of *Mtb* WhiB proteins for DNA binding is not fully understood. It has been previously shown that apo-WhiB3 binds DNA with a low degree of sequence discrimination (Singh *et al*., 2009), which is consistent with a genome-wide study showing the lack of a consensus motif for the WhiB3 DNA binding (Guo *et al*., 2009). Similarly, apo-WhiB1 binds to a large region of promoter DNA rather than a core motif, suggesting a flexible DNA binding specificity (Smith *et al*., 2010). Although, apo-WhiB2 DNA binding is shown to be specific for the *whiB2* promoter (Rybniker *et al*., 2010), a methodical study examining the effect of the redox state of holo- and apo-WhiB2 on DNA binding remains uncharacterized. We show that apo-WhiB4 binds to DNA non-specifically with a preference for GC-rich sequences, interacts with the minor groove of DNA, and represses transcription. While, our *in vitro* data suggest that WhiB4 functions as a non-specific DNA binding repressor, further experiments are needed to conclusively demonstrate the transcriptional regulatory properties of WhiB4 *in vivo*. Nonetheless, the aforementioned characteristics along with a low molecular weight (13.1 kDa) and a highly basic pI (pI = 10.28) of WhiB4 are reminiscent of nucleoid-associated proteins (NAPs) such as HNS, HU and Lsr2 (Colangeli *et al*., 2007; Dillon and Dorman, 2010). The NAPs are promiscuous in their interaction with DNA, but show preference towards AT-rich sequences and minor groove (Dillon and Dorman, 2010).

Interestingly, *Mtb* NAP Lsr2, binds to the upstream regions of *whiB4* and other WhiB4-regulated genes (e.g. *iniB*, *ahpC*, *icl*, etc.), suggesting a potential overlapping role for Lsr2 and WhiB4 in gene expression. However, in contrast to WhiB4, Lsr2 binds *Mtb* DNA with a preference for the AT-rich sequences (AT content ∼ 47%) (Gordon *et al*., 2011). The preference of WhiB4 for DNA fragments with a GC content equivalent to the average *Mtb* genome (i.e. ∼ 65%) suggests a large number of binding sites in the *Mtb* genome, which is contrary to the modest effect of WhiB4 loss on the gene expression. This could simply be due to low endogenous levels of oxidized apo-WhiB4 under the conditions tested. Alternatively, apo-WhiB4 may interact with one or more regulatory proteins (e.g. WhiB6, Rv3249c, Lsr2) that either counterbalance the effect of WhiB4 loss or provide sequence specificity to its DNA binding. Since expression of WhiB4 is induced by nutrient limitation (Betts *et al*., 2002) and enduring hypoxia (Rustad *et al*., 2008), it may well be that under these conditions WhiB4 changes transcriptional pattern by interacting non-specifically with the nucleoid (currently in progress).

The association between oxidative stress and WhiB4 was further substantiated by our data showing repression of *whiB4* in response to CHP treatment and the reported downregulation of *whiB4* expression inside the infected macrophages (Rachman *et al*., 2006). It seems that the regulation of transcription orchestrated by WhiB4 occurs at two levels in response to oxidative stress. Under mild oxidizing conditions (e.g. resting macrophages), sequential degradation of WhiB4 4Fe-4S cluster and subsequent generation of disulphide-linked apo-WhiB4 oligomers result in the transcription repression of stress genes. *Mtb* further regulates the activity of apo-WhiB4 by systematically reducing *whiB4* expression via WhiB4 and/or Lsr2 in response to a gradual increase in oxidative stress (e.g. activated macrophages). This downregulation of WhiB4 can steadily reduce its control on gene expression, necessary for the calibrated derepression of antioxidant and stress genes to avoid excessive virulence for long-term persistence of *Mtb*.

Although our *in vitro* CHP experiment cannot mimic the complexity of redox environment encountered by *Mtb in vivo*, it is tempting to speculate that the uncontrolled expression of antioxidant and stress genes may be partly responsible for the enhanced survival of *Mtb*Δ*whiB4* in macrophages and hypervirulence in the lungs of guinea pigs. Complementation studies confirm that phenotypes are WhiB4-specific. However, contribution of other regulatory factors which either are overexpressed in *Mtb*Δ*whiB4* (WhiB6 and Rv3249c) or modulate *whiB4* expression (Lsr2), should also be examined to fully understand the *in vivo* phenotype of *Mtb*Δ*whiB4* and is the subject of future investigation.

An unexpected observation in this study is that *whiB4* is essential for successful dissemination and/or colonization of *Mtb* in spleen. This finding is difficult to reconcile with the hypervirulence of *Mtb*Δ*whiB4* in the lungs of guinea pigs, unless extrapulmonary dissemination and pulmonary persistence are interrelated. Consistent with this view, it has been shown that dissemination of *Mtb* to extrapulmonary organs precedes the induction of the adaptive immune response in the infected host (Chackerian *et al*., 2002). This suggests that hypervirulence of *Mtb*Δ*whiB4* in the lungs may also be the consequence of weaker stimulation of the protective host response in the spleen due to delayed dissemination of the mutant to the splenic tissues. Alternatively, differences in the environment of spleen and lungs (e.g. O_2_ tension) may have accounted for the tissue-specific phenotype of *Mtb*Δ*whiB4*.

Taken together, our data implicate WhiB4 in regulating oxidative stress response to modulate the virulence of *Mtb*. However, the precise role of WhiB4 in controlling gene expression, dissemination and immune modulation is complex and is a focus of an independent study.

### Ethics statement

This study was carried out in strict accordance with the guidelines provided by the Committee for the Purpose of Control and Supervision on Experiments on Animals (CPCSEA), Government of India. The protocol was approved by the Committee on the Ethics of Animal Experiments of the International Centre for Genetic Engineering and Biotechnology, New Delhi, India (Approval number: ICGEB/AH/2011/2/IMM-26). All efforts were made to minimize the suffering.

## Experimental procedures

### Bacterial strains and growth conditions

*Mtb* H37Rv, *Mtb*Δ*whiB4* and *Mtb whiB4* complemented strains were grown aerobically in inkwell bottles with shaking (150 r.p.m.) at 37°C in 7H9 broth (Difco) or 7H11 agar (Difco) supplemented with 0.2% glycerol, Middlebrook albumin-dextrose-catalase (ADC) enrichment and 0.1% Tween 80 (broth). *E. coli* cultures were grown in LB medium. Antibiotics were added as described earlier (Singh *et al*., 2007).

### Overexpression and purification of WhiB4

The recombinant WhiB4 was purified as N-terminal His-tagged recombinant protein. The entire ORF of *Mtb whiB4* (Rv3681c) was PCR-amplified from *Mtb* H37Rv genomic DNA using gene-specific oligonucleotides pCOLD*whiB4F* and pCOLD*whiB4R* (Table S4). The amplicon was digested with BamHI and HindIII, and ligated into similarly digested His-tag-based expression vector, pCOLD1 (TAKARA BIO, Clontech Laboratories, CA, USA). The resulting plasmid, pCOLD-WhiB4, was then transformed into *E. coli* BL21 (DE3) strain and WhiB4 protein was purified to homogeneity at 15°C by using a His-tag-based affinity purification, as described previously (Singh *et al*., 2009). The *whiB4* gene on pCOLD-WhiB4 was mutated using oligonucleotide-based site-directed mutagenesis approach (Xu *et al*., 1996) to create individual cysteine to alanine substitutions. Sequences of oligonucleotides used to create mutations are shown in Table S4. Resulting clones were verified by sequencing, and the mutant Cys variants of the wild-type WhiB4 were purified as described earlier.

### Fe-S cluster assembly

WhiB4 Fe-S cluster reconstitution was performed under anoxic conditions, monitored by UV-vis spectroscopy and analysed by EPR analysis as described (see SI Experimental procedures).

### EPR spectroscopy

The EDFS EPR measurements were made on an ELEXSYS-E 680 spectrometer (Bruker, Billerica, MA) equipped with an electrically controlled Oxford liquid He transfer line attached to a rectangular type cryostat. EDFS EPR spectra were measured with a two-pulse echo sequence (π/2 − t − π − t − echo). Typically, microwave pulse lengths (tMW) of 16 and 32 ns were used with t = 180 ns. NO-treated WhiB4 was analysed by cw EPR on a perpendicular mode X-band EPR spectrometer operating at 100 kHz modulation frequency and equipped with liquid helium cryostat (Oxford Instruments, Oxon, UK) and a dual mode X-band cavity (Bruker ERA116DM). Field calibration was done by using a standard NMR G meter. EPR was performed at the Department of Biochemistry, University of Alabama at Tuscaloosa, USA.

### Construction of *MtbΔwhiB4*

The allelic replacement of *whiB4* was carried out as described (Bardarov *et al*., 2002). A detailed description is provided in the supplementary information (SI Experimental procedures).

### *In vitro* growth assays

For CHP stress assays, bacteria were grown in 7H9 broth to mid-log phase (OD_600_ of 0.3) and exposed to 25 and 50 µM of CHP (Sigma-Aldrich) for 24 h followed by plating for cfu analysis. For menadione stress assays, bacteria were grown in 7H9 broth at 37°C till mid-log phase (OD_600_ of 0.3) and 10-fold serial dilutions were plated on 7H11 plates containing 0, 20, 40 and 50 µM of menadione (Sigma-Aldrich). The plates were incubated at 37°C for 3–4 weeks and colonies were counted to measure per cent survival.

### Estimation of NAD^+^/NADH and detection of membrane potential

Various strains of *Mtb* were grown in 7H9 medium and subjected to NAD^+^/NADH analysis as previously described (Singh *et al*., 2009). See SI Experimental procedures.

### Microarray hybridization and data analysis

For microarray analysis, the total RNA was extracted from the three biological replicates of aerobically cultured wild-type *Mtb* H37Rv and *Mtb*Δ*whiB4* at an OD_600_ of 0.4 as described (Singh *et al*., 2009). Microarrays were produced, processed and analysed at the Center for Applied Genomics at the Public Health Research Institute, New Jersey (see SI Experimental procedures).

### qRT-PCR

*Mycobacterium tuberculosis* cells were grown till an OD_600_ of 0.4 and RNA was isolated as described (Singh *et al*., 2009). For analysing the influence of CHP on the expression, *Mtb* cells were grown till an OD_600_ of 0.4 and treated with 1 mM CHP for 1.5 h. This was followed by total RNA isolation and qRT-PCR analysis as described (see SI Experimental procedures).

### Preparation of redox modified forms of WhiB4

The redox modified forms of holo-WhiB4 and apo-WhiB4 for the DNA binding reactions were generated as described (see SI Experimental procedures).

### EMSA analysis

For EMSA assays, the promoter fragments (∼ 300–350 bp upstream of the translational start codon) of *ahpC*, *whiB4, pks3, rrnA, espR* were PCR-amplified from the *Mtb* H37Rv genome and the 5′-end was labelled with [γ-^32^P]-ATP (Perkin Elmer) using T4 polynucleotide kinase (MBI Fermentas) according to the manufacturer's instructions. For EMSA analysis with the OxyR binding site in the promoter region of *ahpC*, various oligonucleotides (40 bp) containing *Mtb* and *M. leprae oxyR* binding sites were synthesized as shown in Fig. 7C. Binding reactions were performed in buffer containing 89 mM Tris, 89 mM boric acid and 1 mM EDTA, pH 8.4 in the presence of 0.50 ng of poly dI:dC. The reactions were separated using 4–20% gradient TBE PAGE gels (Bio-Rad). Gels were exposed to autoradiographic film and visualized via phosphorimaging (GE).

### *In vitro* transcription assays

The DNA templates including the putative *whiB4* promoter regions and *rrn*A promoter were prepared by PCR using primers P*whiB4* F1/P*whiB4* R1 and P*rrnA*F1/P*rrnA*R1 respectively (Table S4). The amplicons (50 nM) were pre-incubated with apo-WhiB4 in the transcription buffer (50 mM Tris-HCl, 3 mM magnesium acetate, 0.1 mM DTT, 5% glycerol, 50 µg ml^−1^ BSA and 50 mM KCl) for 30 min at room temperature. Single-round transcription reactions were initiated with the addition of 50 nM RNAP-σ^A^, 100 µM NTPs, 1 µCi [α-^32^P]-UTP, 50 µg ml^−1^ heparin and allowed to proceed at 37°C for 15 min. The reactions were terminated by formamide gel loading dye and then transcripts were resolved on a 10% TBE-urea-PAGE gel (Bio-Rad).

### Construction of the *Msm* strain carrying FLAG-tagged WhiB4

The entire ORF of *whiB4* was PCR-amplified from *Mtb* H37Rv genomic DNA with primers BP-*whiB4F* and BP-*whiB4R*. The PCR product was then cloned downstream of tetracycline responsive *tetRO* regulatory sequences in an *E. coli*-mycobacterial shuttle vector pEXCF to generate pEXCF-*whiB4* using GATEWAY™ Cloning Technology (Invitrogen) as per manufacturer's instructions. The C-terminus of *whiB4* ORF was cloned in frame with the FLAG-tag sequence present in the pEXCF vector. The pEXCF-*whiB4* was then electroporated into *Msm* and transformants were selected on hygromycin. The expression of FLAG-tagged WhiB4 was induced by adding 200 ng ml^−1^ anhydrotetracycline (Cayman chemicals) to the logarithmically grown *Msm* cultures for 4 h at 37°C.

### Non-reducing Western blot analysis

The apo-form of aerobically purified WhiB4 was generated as described earlier. The apo-WhiB4 was either exposed to atmospheric O_2_ or treated with β-ME and resolved by 12% non-reducing SDS-PAGE. Proteins were transferred on to 0.2 µm PVDF membrane and used for Western blot. Western blot analysis was achieved using 1:4000 dilution of anti-His antibody (Qiagen) for 12 h. The blotted membrane was developed with a 1:4000 dilution of peroxidase-conjugated anti-mouse IgG (Cell Signaling) and an enhanced chemiluminescence substrate (GE Amersham).

*Msm* WhiB4 FLAG-tag strain was grown aerobically in flask shaking at 200 r.p.m. till an OD_600_ of 0.5, induced with 200 ng ml^−1^ ATc for 4 h at 37°C, and pelleted. Pellets were resuspended in lysis buffer (300 mM NaCl, 20 mM Na-Phosphate, 10% Glycerol and protease inhibitor, pH 7.5) and sonicated. Sample (30 µg) was added to non-reducing loading dye and separated by non-reducing SDS-PAGE. Proteins were transferred on to a 0.2 µm PVDF membrane and used for Western blot. Western blot analysis was achieved using 1:4000 dilution of anti-FLAG antibody (Sigma-Aldrich) for 12 h and blots were developed as described earlier. For NEM experiment, *Msm* WhiB4 FLAG-tag cells were harvested and suspended in the lysis buffer containing 10 mM NEM for 10 min followed by sonication and non-reducing Western blot analysis.

### Survival of *MtbΔwhiB4* in macrophages

Raw 264.7 macrophages were either non-activated or activated with rIFN-γ (50 U ml^−1^) and LPS (10 ng ml^−1^) for 16 h. Various strains of *Mtb* were added at a multiplicity of infection of 10 to triplicate wells, incubated for 4 h, washed and resuspended in gentamicin-containing fresh DMEM medium. Samples were collected at 0, 2 and 4 days post infection by lysing infected cells with 0.1% SDS. Lysates were diluted in PBS/Tween and plated on 7H10 agar. Colonies were counted after 3–4 weeks at 37°C. Infection of THP-1 human monocytic cell lines was performed as described previously (Kumar *et al*., 2010). Infected cells were cultivated at 5% O_2_ concentration (pO_2_ 36 mmHg) in a humidified incubator (New Brunswick Scientific) and processed for cfu analysis at 0, 2 and 4 days post infection as described earlier.

### Aerosol infection of guinea pigs

Outbred Hartley guinea pigs (∼ 300–400 g body weight) (National Institute of Nutrition, Hyderabad, India) were given a low dose of *Mtb* using a Madison chamber aerosol generation instrument calibrated to deliver 50–100 cfu. Animals were sacrificed (*n* = 5) at 1, 30 and 45 days post infection for determination of organ bacterial burden and histopathology analysis. The statistical significance of the differences between experimental groups was determined by two-tailed, unpaired Student's *t*-test. Differences with a *P*-value of < 0.05 were considered significant.

Histopathology analysis was performed as described previously (Singh *et al*., 2003). Briefly, sections of lungs and spleen were fixed in 10% neutral buffered formalin for embedding in paraffin, sectioning and staining with haematoxylin and eosin. A blinded examination of at least three serial sections from each guinea pig was carried out to evaluate the number of granulomas, inflammation, degree of necrosis and mixed cells infiltrate.
