# Supplementary material for: Mycobacterium tuberculosis WhiB4 regulates oxidative stress response to modulate survival and dissemination in vivo
Source: Mol Microbiol. 2012 Jul 26;85(6):1148–65. doi: 10.1111/j.1365-2958.2012.08165.x (PMC3438311; doi:10.1111/j.1365-2958.2012.08165.x)
Supplement: Supplementary file 1 [file mmi0085-1148-SD1.pdf]

Supporting information

Experimental Procedures

Fe-S cluster assembly: Aerobically purified WhiB4 was completely degassed by using compressed argon gas and anaerobically equilibrated with buffer containing 50 mM sodium phosphate, pH 7.5, and 150 mM NaCl. This was followed by the addition of a 10-fold molar excess Fe(II) chloride, 5 mM DTT, and 2.5 µg of NifS. The reaction was initiated by adding 2 mM L-cysteine and subsequently monitored over time by UV-visible spectroscopy for Fe-S cluster assembly. The reaction was terminated by size-exclusion chromatography to remove low molecular polymers. For reduction, a sample of fully reconstituted WhiB4, after passing through a size-exclusion column, was treated with 5 mM DTH and analyzed by UV-visible spectroscopy. For EPR spectroscopy, reconstituted WhiB4 before (oxidized) and after (reduced) DTH treatment was transferred to EPR tubes and immediately frozen in liquid nitrogen. To study the effect of O_2 on Fe-S cluster stability, a sample of fully reconstituted WhiB4 was purified by size-exclusion chromatography, transferred to an anaerobic cuvette, and exposed to air by opening the cap of the cuvette and mixing by pipetting for 2 min. The cuvette was then sealed and monitored by UV-visible spectroscopy (DU800; Beckman Coulter, Fullerton, CA). For EPR analysis at liquid helium temperature, reconstituted WhiB4 was transferred to an EPR tube 5 and 30 min after air exposure and immediately frozen in liquid nitrogen. For NO exposure, anaerobically reconstituted WhiB4 was treated inside the anaerobic glove box

with aliquots of freshly prepared proline NONOate (Cayman Chemicals, Ann Arbor, MI). Half-life of proline NONOate is 1.8 sec at pH 7.4. Aliquots of WhiB4 were placed in an anaerobic cuvette and titrated by injection with aliquots of a 2.5 mM stock solution of proline NONOate. Samples were then transferred to EPR tubes and immediately frozen in liquid nitrogen.

Construction of *Mtb∆whiB4*

Complementary oligonucleotides were used to amplify the DNA fragment containing *whiB4* from the *Mtb* genome. An internal fragment of the *whiB4* ORF was replaced by a *lox*P-chloramphenicol:hygromycin-*lox*P cassette and cloned into pYUB572 (Bardarov *et al.*, 2002) to create pYUB*whiB4*. The allelic replacement of *whiB4* was carried out as described (Bardarov *et al*., 2002), and disruption was confirmed by PCR and Southern blot analysis. The disruption eliminated amino acids 33 to 86 of the 118 amino acid WhiB4 protein. To construct the *whiB4* complemented strain, the *whiB4* ORF, along with its native promoter (~500 bp sequence upstream to ATG) and the ribosomal binding site were cloned into the *E.coli*-mycobacterial shuttle integrative vector pCV125 (Alland *et al.*, 2000) to generate pCV125:*whiB4*. The *Mtb∆whiB4* strain was transformed with pCV125:*whiB4* and the expression of *whiB4* was confirmed in the transformants by qRT-PCR (Table S2).

Microarray hybridization and data analysis

Microarrays used in this study were produced and processed at the Center for Applied Genomics at Public Health Research Institute, New Jersey. These microarrays consist of 4,295 70-mer oligonucleotides representing 3,924 ORFs

from *Mtb* H37Rv (http://www.sanger.ac.uk) and 371 unique ORFs from strain CDC1551 (http://www.tigr.org) that are absent in the *Mtb* H37Rv strain. For microarray analysis, total RNA was extracted from the three biological replicates of wild type *Mtb* H37Rv and *MtbΔwhiB4* at an OD_{600} of 0.4 as described (Kumar *et al.*, 2008). Briefly, 1.5 μg of RNA was reverse transcribed with random hexamers (Invitrogen) and the resulting complementary DNA was labeled with Cy3-dUTP or Cy5-dUTP (PerkinElmer), and competitively hybridized to whole genome arrays. Hybridization was performed overnight. After washing the arrays were dried by centrifugation (100 x *g*, 2 min) and scanned. The detailed labeling and hybridization protocol can be obtained at http://www.cag.icph.org/downloads_page.htm#LabelingProtocols.

Scan Protocol:

The arrays were scanned with a GenePix4200AL scanner (Molecular Devices, Sunnyvale, CA). The images were processed using GenePix 6.1 and the resulting text files were exported to Microsoft Excel.

Data Process:

The chips were normalized by the print-tip Lowess method (Pang *et al.*, 2007). The data were filtered by removing all the spots that were below the background noise or flagged as 'bad'. Also, spots were flagged if they were not present in two out of three replicates. The images were processed using GenePix pro 6.1 software. The ratio of the mean median intensity of Cy5 over the mean median intensity of Cy3 was determined for each spot and the fold change values were calculated. The data was further processed through a modified t-test (SAM;

significance analysis of microarrays) (Tusher *et al.*, 2001) using MEV software (Saeed *et al.*, 2003). We set the false discovery rate to *zero* for obtaining tightest possible data. The microarray data discussed in this manuscript have been deposited in NCBI's Gene Expression Omnibus and are accessible through GEO series accession number GSE37840.

qRT-PCR analysis

Mtb cells were grown till an OD_{600} of 0.4 and RNA was isolated as described (Kumar et al., 2008). First-strand cDNA synthesis was performed using 500 ng of the total RNA with iScript Select cDNA Synthesis Kit (Bio-Rad) using random oligonucleotide primers. PCR was performed using gene specific primers (Table S4). Gene expression was analyzed with real-time PCR using iQ^{TM} SYBR Green Supermix (Bio-Rad) and a CFX96 RT-PCR system (Bio-Rad). Data analysis was performed with the CFX Manager^{TM} software (Bio-Rad). PCR efficiencies were normalized to obtain accurate expression levels. For comparison between wt *Mtb*, *MtbΔwhiB4*, and the *whiB4* complemented strains the induction ratio for each gene was normalized to *Mtb* 16S rRNA expression.

Estimation of NAD^+/NADH and detection of membrane potential

Briefly, *Mtb* cells were rapidly harvested and resuspended in 0.2 M HCl (NAD^+ determination) or 0.2 M NaOH (NADH determination). Pyridine nucleotide extraction was further facilitated by heating samples at 80°C for 20 min. Samples were then neutralized with 100 µl of base (0.1 M NaOH) or 100 µl of acid (0.1 M HCl) for NAD^+ and NADH, respectively. The NAD^+/ NADH ratio was determined by cycling assay using 96 wells format. The nucleotide cycling cocktail contained

2 ml 1 M bicine buffer (pH 8.0), 2 ml 100% ethanol, 2 ml 40 mM EDTA (pH 8.0), 2 ml 4.2 mM thiazolyl blue tetrazolium bromide (MTT), and 4 ml 16.6 mM phenazine ethosulfate (PES). The required wells in the plate were filled with 100 µl of cycling cocktail and 90 µl of cell extract. The mixture was mixed with 10 µl alcohol dehydrogenase (Sigma-Aldrich; 500 U/ml). The reduction of MTT by alcohol dehydrogenase using NADH was monitored at 565 nm each minute for 10 min. The intensity of the reaction product color is directly proportional to the NAD^+/NADH concentrations in the sample. NAD^+ and NADH standards were included in separate wells to determine concentrations. The data reported is the result of 3 independent experiments assayed in triplicate. Assaying for membrane potential was performed on similarly cultured cells using the BacLight bacterial membrane potential kit (Invitrogen, Life Technologies) as described (Rao *et al.*, 2008). Briefly, cells were diluted to an OD_{600} of 0.05 in Dubos medium and 1 ml of cells were stained using 3 µM of $DiOC_2$ for 15 min in the presence or absence of 25 µM CCCP. The cells were fixed using 2% paraformaldehyde and analyzed for fluorescein and texas red dye using flow cytometry.

Preparation of redox modified forms of WhiB4

The redox modified forms of holo-WhiB4 for the DNA binding reactions were generated by anaerobically reassembling the Fe-S cluster as described in the earlier section. Holo-WhiB4 was separated from low molecular weight polymers by size exclusion chromatography and DNA binding was performed in the anaerobic glove box as described (Singh *et al.*, 2009). Apo-WhiB4 was

generated as previously described (Alam *et al.*, 2007). Reduced apo-WhiB4 was generated by addition of 300 mM DTT. Oxidized apo-WhiB4 was generated by removal of DTT from the apo-WhiB4 by size exclusion chromatography followed by treating the samples either with 50 mM diamide or exposure to air for 2 h.

SI Notes

SI note 1:

A) Electron paramagnetic resonance (EPR) is a technique analogous to NMR, allowing the study of paramagnetic compounds (i.e. chemical species that have one or more unpaired electrons) such as free radicals or transitional metals. EPR spectroscopy involves the measurement of the absorption of electromagnetic radiation contributed by a paramagnetic system positioned in a fixed magnetic field. Thus, in EPR, it is the electron spins that are excited rather than the spins of atomic nuclei. EPR gives information on the environment of the unpaired electron, and can help to decide the extent to which electrons are delocalized over the ligands. Since, Fe-S clusters often display paramagnetism ($[4Fe\text{-}4S]^{1+}$, $[4Fe\text{-}4S]^{3+}$, $[3Fe\text{-}4S]^{1+}$ etc.), EPR is a powerful tool for studying Fe-S clusters, their core oxidation state, and kinetics in response to redox signals such as O_2 or NO.

B) UV-visible spectroscopy or absorption spectroscopy is based on detecting colors in the ultra-violet spectral region. The Fe-S clusters give strong, distinctive spectra because they often display intense yellowish/brownish color. This technique provides limited information about the redox state of an Fe-S cluster. UV–vis spectroscopy is most often used in cluster chemistry as a means

to determine reaction kinetics, such as the rate of Fe-S cluster reconstitution (observed by the development of yellowish brown color), degradation of Fe-S cluster by O_2 (loss of yellowish brown color), and reaction between NO donor and Fe-S cluster protein (change of color to yellowish green).

SI note 2:

In vitro transcription assays are widely used to understand the mechanisms of gene regulation. Until now, *in vitro* transcription assays for studying gene regulation in *Mtb* were generally performed using holo-RNA polymerase (holo-RNAP) prepared by the reconstitution of *E. coli* core RNAP (i.e., RNAP lacking a sigma factor) with excess of purified sigma factor derived from *Mtb* (Song *et al.*, 2008) The holo-RNAP containing both *E. coli* and mycobacterial components may have altered transcription properties, making these assays prone to artifacts. Recently, native holo-RNAP purified from *Mycobacterium smegmatis* (*Msm*) was extensively used to perform *in vitro* transcription assays (Smith *et al.*, 2010). However, since *Msm* contains 26 putative sigma factors, native holo-RNAP was found to exist as a complex mixture of different holoenzymes harboring distinct sigma factors and showed poor activity and low promoter specificity (China and Nagaraja, 2010). The recently described *in vivo* reconstitution of holo-RNAP with a specific mycobacterial sigma factor promises to overcome these limitations (China and Nagaraja, 2010). This *in vivo* expression technology allowed precise assembly of core RNAP with the overexpressed sigma factor inside *Msm* in stoichiometric amounts. The RNAP-σ^A purified by the *in vivo* expression technology showed high activity and specificity

towards σ^A-dependent promoters (China and Nagaraja, 2010). Using the highly efficient *in vivo* reconstituted RNAP-σ^A, we have demonstrated the influence of apo-WhiB4 on transcription from *whiB4* and *rrnA* promoters by performing single-round transcription assays. Our selection of RNAP-σ^A for transcription assays was guided by the fact that another WhiB family member (WhiB3) interacts specifically with the σ^A (Steyn *et al.*, 2002).

SI note 3: Promoter sequences of *rrnA* and *whiB4*

P*rrnA*

-35 -10

GACGGTCACCTATGGATATCTATGGATGACCGAACCTGGTC**TTGACT**CCATTGCCGGATTTG**TATTAG**A

⬇

CTGGCAG**G**GTCGCCCCGAAGCGGGCGGAAACAAGCAAGCGTGTTGTTTGAGAACTCAATAGTGTGTTTGGG

TGGTTTCACATTTTTGTTGTTATTTTTGGCCATGCTCTTGATGCCCCGTTGTCGGGGGCGTGGCCGTTTGT

TTTGTCAGGATATTTCTAAATACCTTTGGCTCCTTTTCCAAAGGGAGTGTTTGGGTTTTGTTTGGAGAGTT

P*whiB4*

AGTAGCGACCTCCTGTCACTCCACCGTGCCGCTCGGTCCAATAGAATCTTTCCGCGGGCGGGTGAGTCACT

-10 ⬇

GCGGGATCGGGGCGGG**CGCGAT**ATTTGCGG**C**TGCGTCACTGGTCAGACCCCGTAAACGGGACCGAAGCGG

CGGCATTTCCCCCGGCCGATCCAGACAAGACGGCACGCTCGTCCCAAAAAAGCGTTATCGTACTGTTGCGC

AATTGGCACACGACCACCTGACTTATACAGATCAGTGAGATACAGGTAACACCGATGTGCATGGTGTGGCG

TCGCTCGCAGCATCGGCTGTTTCCGAGGAGGGCGGTCGCACACGGCGGCCGGGAGGAAGGGATCAGCTCGT

GTCAGGAACCCGTCCAGCC

The sequences of *rrnA* and *whiB4* promoter fragments used for *in vitro* transcription assays are displayed above. The primers used for PCR amplification are underlined for both the genes. The σ^A-dependent -35 and -10 sequences for *rrnA* are shown in bold letters and the transcriptional start point (TSP) by a solid arrow (Gonzalez-y-Merchand *et al.*, 1996, Verma *et al.*, 1994). A

transcript of 206 bp is expected upon transcription of the *rrnA* amplicon, which corroborates to the size observed in figure 6B.

To predict the promoter and TSP for *whiB4*, we analyzed the upstream region of *whiB4* ORF by the Artificial Neural Networks Promoter Prediction (ANNPP 2.2, http://www.fruitfly.org/seq_tools/~promoter.html) program (Kalate *et al.*, 2003). The ANNPP is based on error-back-propagation (EBP) algorithm and has been successfully utilized with high efficiency (~97% prediction capability) to predict promoters and TSPs of several mycobacterial genes (Kalate *et al.*, 2003). The ANNPP analysis of the *whiB4* promoter region revealed the presence of a putative TSP (as indicated by a solid arrow) at 250 bp upstream of translational start site of WhiB4. Based on the TSP identified by ANNPP, a transcript of ~ 260 bp is expected upon transcription of the *whiB4* amplicon, which is in agreement with the size obtained in the i*n vitro* transcription assays (Fig. 6A). However, the TSP and -10 motif of *whiB4* need experimental verification and are a focus of an independent study.

Supplementary figures

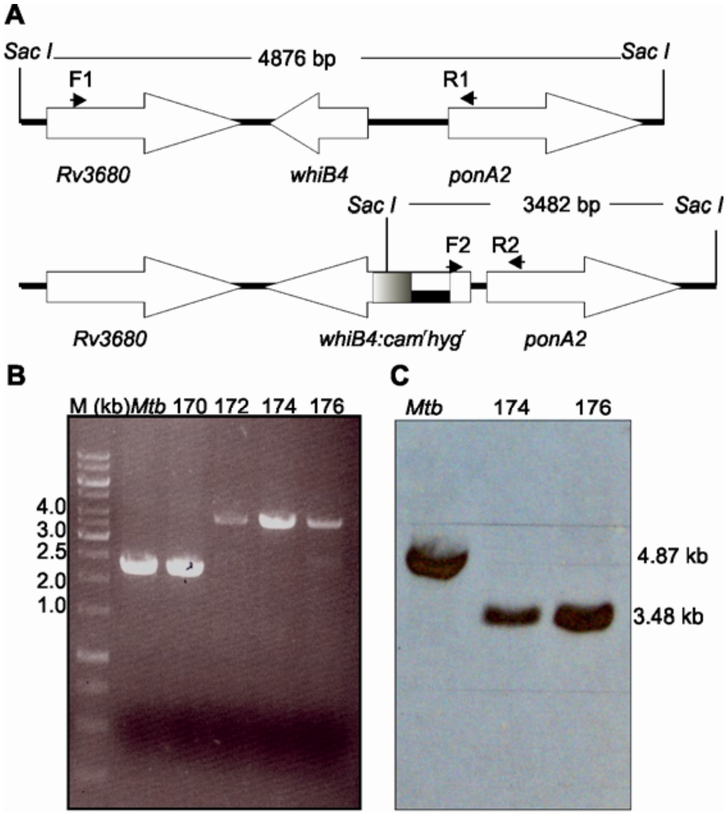

Fig. S1: Disruption of wt *Mtb* H37Rv *whiB4* loci. (A) A schematic representation of *whiB4* loci in the genome of *Mtb* H37Rv before (upper panel) and after (lower panel) replacement with *whiB4:cam^{r}hyg^r* fragment. (B) Four hygromycin resistant colonies (170, 172, 174, and 176) were examined by PCR using F1 and R1 primers (shown in A). An increase in the size of the PCR fragment in the case of 172, 174, and 176 is consistent with the disruption of the wt *whiB4* allele. (C) The disruption of *whiB4* was further confirmed by performing Southern blot analysis of clones 174 and 176. The primers F2 and R2 (shown in A) were used to amplify a 623 bp region encompassing the 3’-end of *whiB4* ORF and flanking sequences to be used as a probe. This fragment was used to probe *Sac* I digested chromosomal DNA isolated from *Mtb* cells and separated on a

0.8% agarose gel using Amersham ECL Direct Nucleic Acid Labeling and Detection Systems. As predicted in figure A, and shown in C, insertional inactivation of *whiB4* resulted in the replacement of a 4.87 kb *Sac* I fragment from wt *Mtb* with a 3.48 kb fragment in the *whiB4* mutants. Appearance of a shorter fragment in the case of *whib4* mutants results from an additional *Sac* I site in the chloramphenicol:hygromycin resistance cassette.

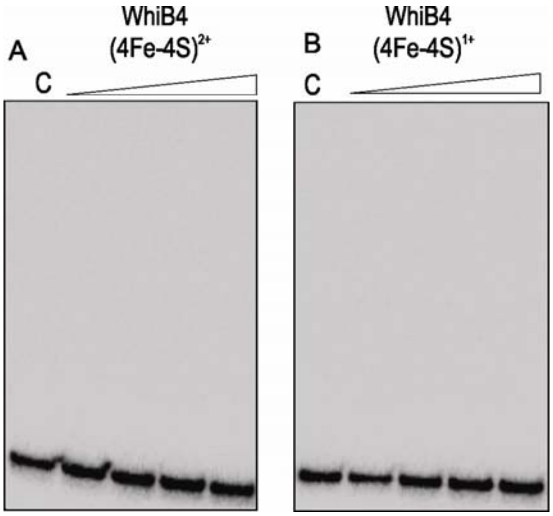

Fig. S2: DNA binding activity of holo-WhiB4. The $[4Fe\text{-}4S]^{2+}$ cluster in WhiB4 was reconstituted under anaerobic conditions and confirmed via UV-visible spectroscopy (see *Experimental procedures*). Increasing concentrations (0.2, 0.4, 0.8, 1 μM) of (A) oxidized ($[4Fe\text{-}4S]^{2+}$) and (B) DTH reduced ($[4Fe\text{-}4S]^{1+}$) holo-WhiB4 were assayed for DNA-binding to 0.2 nM of γ-^{32}P labeled *ahpC* promoter under anaerobic conditions. C: DNA binding in the absence of WhiB4 in each panel.

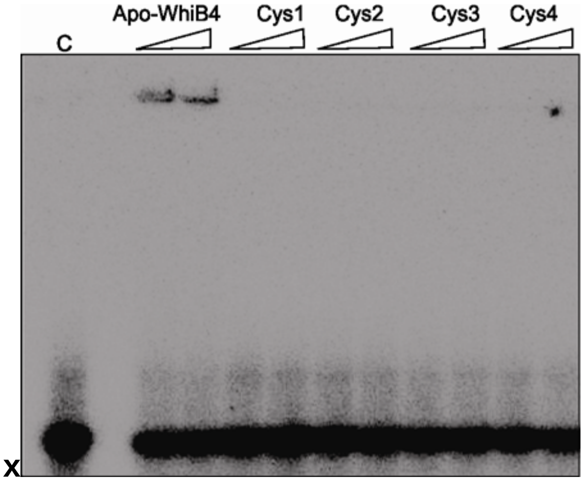

Fig. S3: DNA binding activity of apo-WhiB4 Cys →Ala mutant proteins. Wild type apo-WhiB4 and the Cys mutant (Cys1, Cys2, Cys3, and Cys4) proteins were prepared and oxidized by diamide as described in the *Experimental procedures*. EMSA reactions were performed with 0.2 nM of γ-^{32}P labeled *ahpC* promoter DNA fragment and 200 and 400 nM of wild type and mutant proteins. C: DNA binding in the absence of WhiB4.

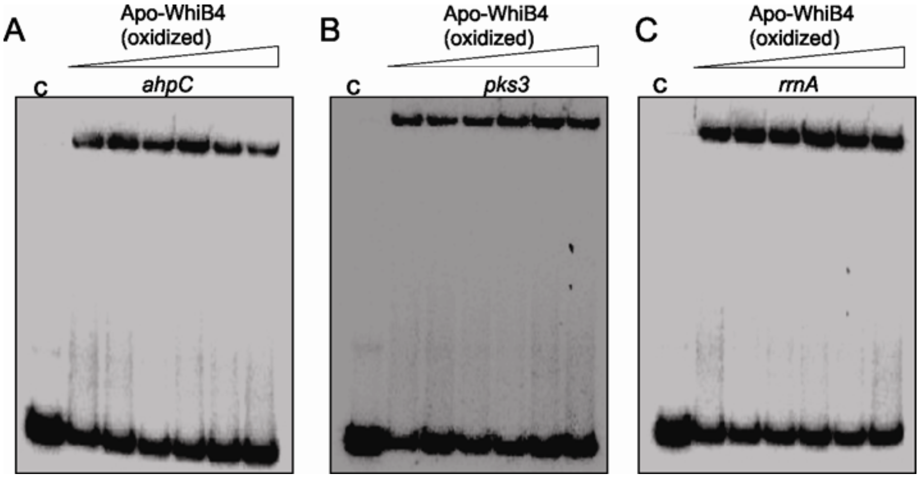

Fig. S4: WhiB4 binds DNA in a sequence-independent manner. EMSAs were performed with 0.2 nM of ^{32}P labeled *(A) ahpC* (WhiB4-regulated), (B) *pks3* (non-regulated), and (C) *rrnA* (non-regulated) promoter DNA fragments. The

concentrations of oxidized apo-WhiB4 used were 1, 1.5, 2, 2.5, 3.0, 3.5 μM. C: DNA binding in the absence of WhiB4 in each panel.

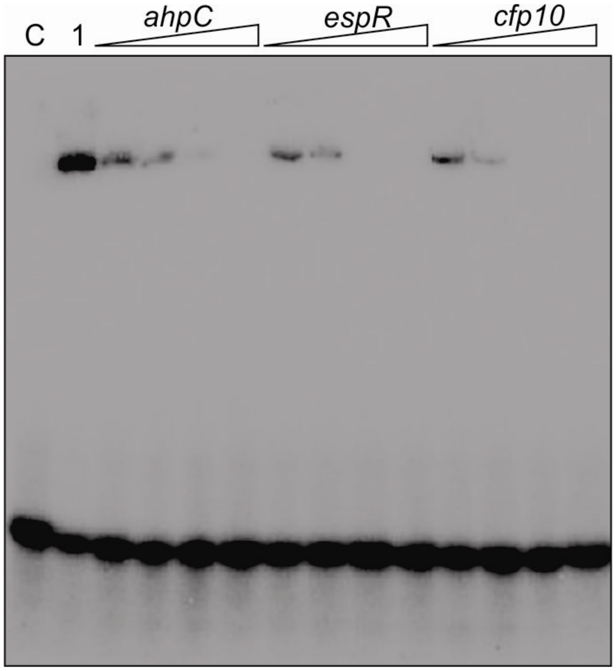

Fig. S5: *espR* and *cfp10* promoters effectively compete for binding of WhiB4 to the *ahpC* promoter. Competition assays using *ahpC* (WhiB4-regulated), *espR* (non-regulated), and *cfp10* (non-regulated) promoter fragments. Lane 1: WhiB4:*ahpC* promoter fragment complex. DNA binding was competed using increasing concentrations (20, 30, 40 and 50-fold molar excess) of either unlabeled *ahpC* or *espR* or *cfp10* promoter DNA C: DNA binding in the absence of WhiB4. The *ahpC* fragment used in the assay is an ~40 bp oligonucleotide containing OxyR binding motif present in the promoter region of *Mtb ahpC*.

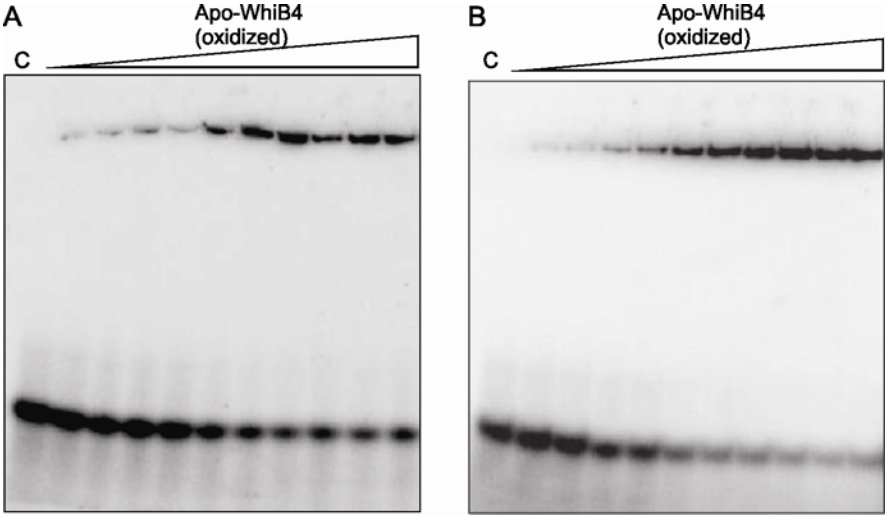

Fig. S6: Apo-WhiB4 binds to the GC-rich M3 (68%) and M4 (85%) DNA fragments in a concentration- dependent manner. EMSAs were performed with 0.2 nM of γ-^{32}P labeled (A) M3 and (B) M4 DNA fragments. The concentrations of oxidized apo-WhiB4 were 0.1, 0.2, 0.3, 0.4, 0.5, 0.6, 0.7, 0.8, 0.9, 1 μM. C: DNA binding in the absence of WhiB4 in each panel. The K_d for M3 and M4 was calculated by performing the densitometry analysis of free and protein bound DNA.

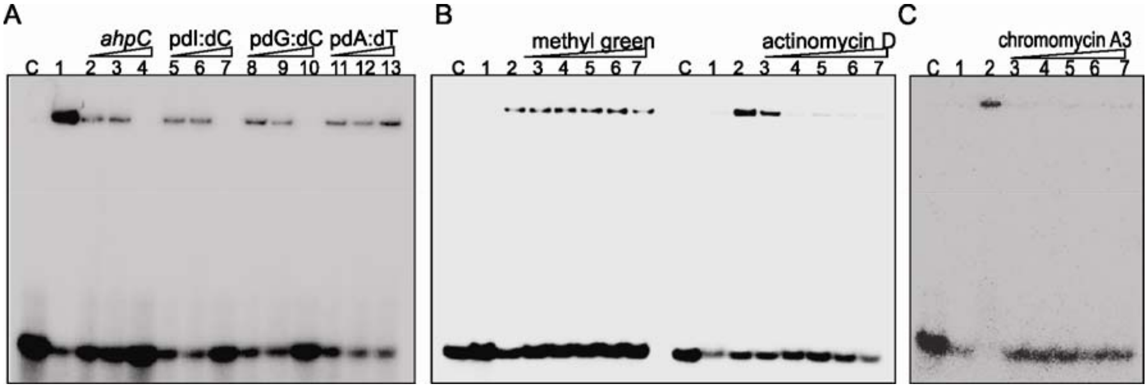

Fig. S7: (A) Competition assays using poly (dI:dC), poly (dG:dC), and poly (dA:dT). Lane 1: WhiB4:*ahpC* promoter fragment complex and DNA binding was competed using 10, 20, and 50-fold molar excess of either unlabeled *ahpC*

(lanes 2, 3, and 4) or poly dI:dC (lanes 5, 6, and 7) or poly dG:dC (lanes 8, 9, and 10) or poly dA:dT (lanes 11, 12, and 13). **(B and C) WhiB4 binds to the minor groove of *ahpC* promoter fragment.** Apo-WhiB4 binding to the *ahpC* promoter complex was competed out by the DNA binding drugs, methyl green (major groove binder), actinomycin D (minor groove binder), and chromomycin A3 (minor groove binder). Lane 1: DNA and 1 mM of either methyl green or actinomycin D or chromomycin A3, lane 2: apo-WhiB4:*ahpC* promoter complex, lanes 3 to 7: DNA binding was competed using increasing concentrations (0.2 mM, 0.4 mM, 0.6 mM, 0.8 mM, and 1 mM) of methyl green or actinomycin D or chromomycin A3. C: DNA binding in the absence of WhiB4 in each panel. Since chromomycin A3 prefers minor groove of the GC-rich DNA (Zihlif *et al.*, 2010), GC-rich *ahpC* fragment M4 was used for the competition assays.

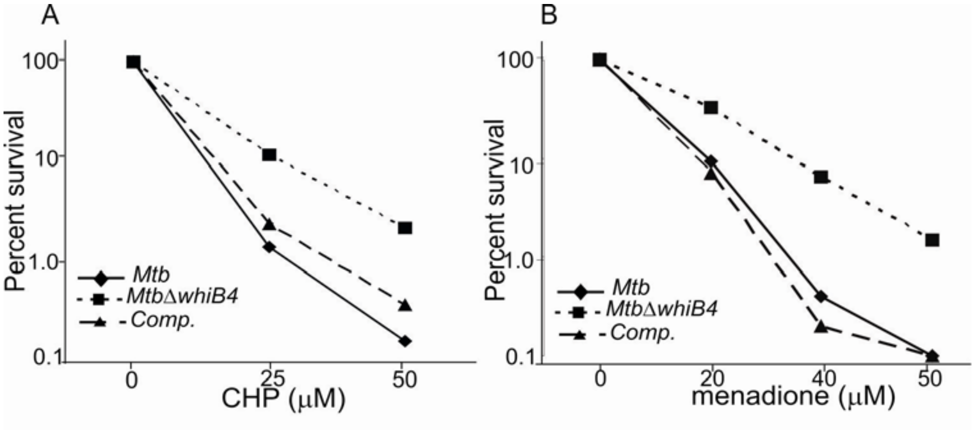

Fig. S8: WhiB4 modulates oxidative stress survival in *Mtb*

(A) Exponentially growing cultures of *Mtb* were synchronized to an OD_{600} of 0.3 and exposed to 25 μM and 50 μM of cumene hydroperoxide (CHP). At 0 hr and 24 hr post exposure, the viable counts were determined to calculate percent survival. The data presented is the average (± standard deviation) of three

independent experiments performed in triplicate. (B) 10-fold serial dilutions of exponentially growing cells were plated on 7H11 plates containing 0, 20, 40, and 50 μM of menadione. The plates were incubated at 37°C for 3-4 weeks till visible colonies appeared. The number of colonies on the menadione-containing medium was reported as the percentage of the number of colonies on the menadione-free medium. The data presented is the average (± standard deviation) of two independent experiments performed in triplicate. The error bars are smaller than the size of the data points symbols.

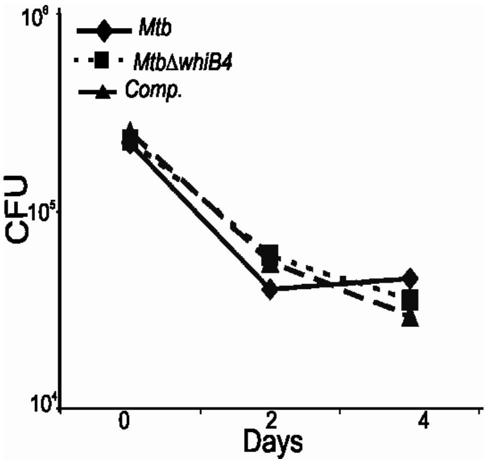

Fig. S9: Intracellular growth of *MtbΔwhiB4* inside resting Raw 264.7 macrophages. Resting Raw264.7 cells were infected with *Mtb* strains at a MOI of 10 and growth was monitored over time by CFU analysis. The data shown was the average of three experiments performed in triplicate.

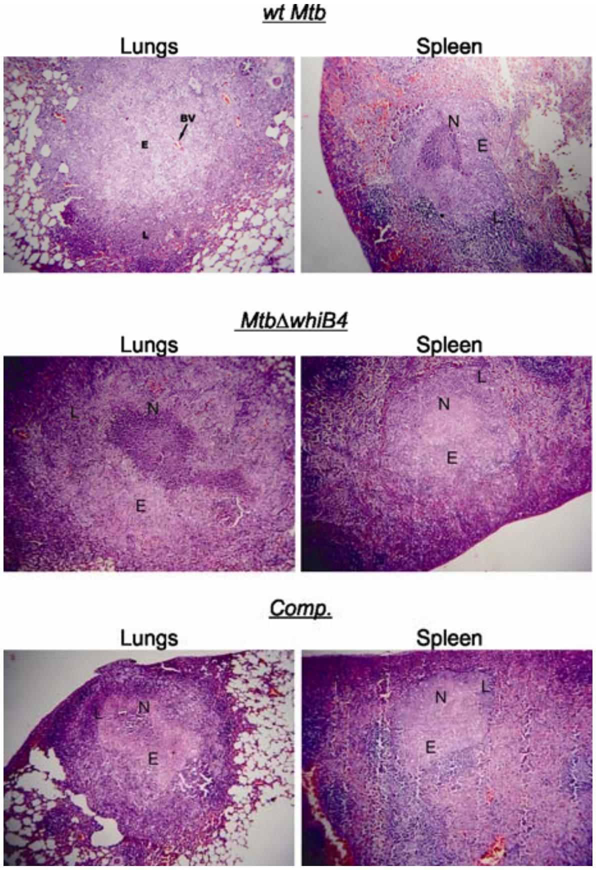

Fig. S10: *In vivo* pathology caused by *MtbΔwhiB4*. Hematoxylin and eosin stained lung and spleen sections (45 days post-infection) from guinea pigs infected with wt *Mtb*, *MtbΔwhiB4* and complemented strain. The pathology sections show granulomas containing areas of necrosis (N), epithelioid cells (E), blood vessel (BV) and lymphocytes (L). All images were taken at 4X magnification.

Table S1. Microarray analysis of genes found to be up-regulated in *Mtb∆whiB*4 compared to wt *Mtb*.

Genes	Rv No.	Fold change	Description
nrdB	Rv0233	1.3 ± 0.02	Ribonucleoside - diphosphate reductase B chain
iniB	Rv0341	1.56 ± 0.05	Isoniazid inducible protein
icl	Rv0467	1.31 ± 0.16	Isocitrate lyase
CHP	Rv0692	1.67 ± 0.15	Conserved hypothetical protein
pqqE	Rv0693	1.38 ± 0.07	Coenzyme pQQE synthesis protein E
lldD1	Rv0694	1.24 ± 0.01	L-lactate dehydrogenase-1
fabD	Rv2243	1.30 ± 0.06	Malonyl CoA:AcpM acyltransferase
acpM	Rv2244	1.36 ± 0.03	Meromycolate extension acyl carrier protein M (ACPM)
kasA	Rv2245	1.38 ± 0.08	Beta-Ketoacyl ACP synthase A
kasB	Rv2246	1.44 ± 0.26	Beta-Ketoacyl ACP

			synthase B
ahpC	Rv2428	1.30 ± 0.02	Alkylhydroperoxidase C
ahpD	Rv2429	1.30 ± 0.26	Alkylhydroperoxidase D
ppsA	Rv2931	1.51 ± 0.14	Phenophthiocerol synthesis Type I polyketide synthase
Rv3249c	Rv3249c	1.64 ± 0.11	TetR family Transcriptional regulator
rubB	Rv3250c	1.56 ± 0.02	Rubredoxin B
rubA	Rv3251c	1.69 ± 0.01	Rubredoxin A
alkB	Rv3252c	1.70 ± 0.03	Alkane1-monoxygenase
whiB4	Rv3681c	1.96 ± 0.05	Transcription regulator (WhiB family)
whiB6	Rv3862c	1.69 ± 0.04	Transcription regulator (WhiB family)
Rv3865	Rv3865	1.30 ± 0.02	Conserved Hypothetical protein

PE35	Rv3872	1.38 ± 0.05	PE-family protein
PPE68	Rv3873	1.44 ± 0.09	PPE-family protein
PPE19	Rv1361c	-2.53 ± 0.22	PPE-family protein

ORF numbers, gene names, and gene products are as annotated in the work of Cole et al. (Cole *et al.*, 1998) or on the website http://www.tigr.org.

Table S2: qRT-PCR analysis of genes found to be regulated by WhiB4.

Genes	*Fold change (Mtb∆whiB4/wt Mtb)*	*Fold change (comp./wt Mtb)*
pqqE	5.6±0.9	1.12±0.04
ahpC	3.8±0.6	0.97±0.02
ahpD	4.2±1.2	0.86±0.01
rubA	6.5±1.6	1.24±0.01
rubB	4.0±0.7	0.91±0.08
Rv3249c	4.8±0.3	0.67±0.02
whiB6	4.4±0.9	0.89±0.03
pks3	1.1±0.03	0.97±0.01
cfp10	0.9±0.01	1.13±0.03

Total RNA was isolated from logarithmically grown cells of wt *Mtb*, *Mtb∆whiB4*, and *whiB4* complemented (*comp.*) strain and the expression of various genes was analyzed by qRT-PCR. The expression of each gene was normalized to 16S rRNA expression. Data shown is the average (± standard error of mean) of two independent experiments performed in triplicate. As a control, we compared the

expression of unrelated genes such as *pks3* and *cfp10* among various *Mtb* strains.

Table S3: qRT-PCR analysis of genes regulated by WhiB4 upon exposure to CHP.

Genes	*Fold change (CHP treated wt Mtb/wt Mtb)*	*Fold change (CHP treated Mtb∆whiB4/ Mtb∆whiB4)*	*Fold change (CHP treated comp./comp.)*
Rv0692	2.9 ±0.17	7.2 ±1.54	1.8 ±0.09
ahpC	2.3 ±0.10	3.8 ±0.98	2.5 ±0.17
ahpD	2.2±0.21	4.7±0.86	2.8±0.07
rubA	1.8±0.16	3.5±0.61	1.6±0.23
Rv3249c	3.4±0.32	5.1±1.87	2.7±0.62
whiB6	4.6±0.11	16.9±2.98	5.8±0.32
whiB4	- 4.4±0.11	1.6±0.76	- 3.4±0.17
pks3	0.9±0.07	0.7±0.13	1.1±0.01
cfp10	0.9±0.03	1.2±0.19	0.9±0.23

Logarithmically grown cells (OD_{600} of 0.4) of wt *Mtb*, *Mtb∆whiB4,* and *whiB4* complemented (*comp.*) were divided in two equal parts. One part was treated with 1 mM of CHP for 1.5 h and the other part was left untreated and the total RNA was isolated from each part. The expression of various genes was analyzed

by qRT-PCR. For comparison between the untreated and CHP treated wt *Mtb*, *MtbΔwhiB4,* and *whiB4* complemented strains, the induction ratio of each gene was normalized to *Mtb* 16S rRNA. To check the expression of *whiB4* in *MtbΔwhiB4*, we amplified the initial 90 bp of the truncated *whiB4* transcript. Data shown is the average (± standard error of mean) of three independent experiments performed in triplicate.

Table S4: Sequences of primers used in this study.

Gene Name	Primer Sequence (for qRT-PCR)
Rv0692	F: 5’ CGCGCTGCTCTATCACTTCG 3’<br>R: 5’ GACTGTGACCAGGATCCGTA 3’
ahpC	F: 5’ TTTGGCCGAAAGACTTCACG 3’<br>R: 5’ GACATCAAGCGCGAACTCAG 3’
ahpD	F: 5’ CGAAATCCGCAGGTATTAGC 3’<br>R: 5’ CATACCGAAAGCCAACTTCG 3’
rubA	F: 5’ CAACGGTGATGCCAGGGAAG 3’<br>R: 5’ CAAATTCCCGACGACTGGTG 3’
rubB	F: 5’ GTATCCAATGCGGCTTTGAG 3’<br>R: 5’ GATTGCGGTGCGGCAAAGTC 3’
Rv3249c	F: 5’ GCCGGCAGACCATCTACAAC 3’<br>R: 5’ CGATTAGTGGACAACGTCCA 3’
whiB6	F: 5’ CGCAAGATCCCGATCGTTGG 3’<br>R: 5’ CGTAATTCCCGAATCAGGCC 3’

pks3	F: 5’ GATTCACCGGCACCAGTAAC 3’<br>R: 5’ GCAAGAGCGAGATCGCTTTC 3’
cfp10	F: 5’ AGTCGACGGCAGGTTCGTTG 3’<br>R: 5’AGGCGCTGTCCTCGCAAATG 3’
whiB4	F: 5’ CAGCCGCTCGAAGGACTAAC 3’<br>R: 5’ ATCCGCTCTTCCGCGTCTAC 3’
16s rRNA	F: 5’ TTGACGGTAGGTGGAGAAGA 3’<br>R: 5’ CGCAAGGCTAAAACTCAAAGG 3’
**Primer Name**	**Primer Sequence (*whiB4* cloning and site-directed mutagenesis)**
pCOLD*whiB4F*<br>pCOLD*whiB4R*	5’ GATACGGATCCGTGTCAGGAACCCGTCCAG 3’<br>5’ ACCGGCACCGCCGGATAGAAGCTTAGTATC 3’
pCOLD*whiB4Cys1F*<br>pCOLD*whiB4Cys1R*	5’TATCCAAGGCGCTGGCCCGGACTACCGACC3’<br>5’ GGGTATCCAAGGCGCTGGCTCGGACTACCG 3’
pCOLD*whiB4Cys2F*<br>pCOLD*whiB4Cys2R*	5’CAAGGCCGCGGTGATCGCCCGTCACTGTCCGGTAATG3’<br>5’CAAGGCCGCGGTGATCGCCCGTCACTGTCCGGTAATG3’
pCOLD*whiB4Cys3F*<br>pCOLD*whiB4Cys3R*	5’ TGATCTGCCGTCACGCTCCGGTAATGCAAG 3’<br>5’ TGCATTACCGGAGCGTGACGGCAGATCACC 3’
pCOLD*whiB4Cys4F*<br>pCOLD*whiB4Cys4R*	5’ GTCCGGTAATGCAAGAGGCTGCGGCAGATG 3’<br>5’ GGTAATGCAAGAGGCTGCGGCAGATGCGCT 3’
P*whiB4F*<br>P*whiB4R*	5’AGTAGCGACCTCCTGTCAC 3’<br>5’GGCTGGACGGGTTCCTGAC 3’

P*rrnAF*<br><br>P*rrnAR*	5’ GACGGTCACCTATGGATATCTATG 3’<br><br>5’ AACTCTCCAAACAAAACCCAAACACTCC 3’
BP-*whiB4F*<br><br>BP-*whiB4R*	5’ggggacaagtttgtacaaaaaagcaggctctaTGTCAGGAACCCGTC3’<br><br>5’ggggaccactttgtacaagaaagctgggtcTCCGGCGGTGCCGGTG3’

BP- whiB4 primers contain attB recombination sites (indicated by the lower case letters) required for the GATEWAY^{TM} Cloning strategy.

References:

Alam, M. S., Garg, S. K. and Agrawal, P. (2007) Molecular function of WhiB4/Rv3681c of *Mycobacterium tuberculosis* H37Rv: a [4Fe-4S] cluster co-ordinating protein disulphide reductase. *Mol Microbiol* **63:** 1414-1431.

Alland, D., Steyn, A. J., Weisbrod, T., Aldrich, K. and Jacobs, W. R. Jr. (2000) Characterization of the *Mycobacterium tuberculosis iniBAC* promoter, a promoter that responds to cell wall biosynthesis inhibition. *J Bacteriol* **182:** 1802-1811.

Bardarov, S., Bardarov Jr, S. Jr., Pavelka Jr, M. S. Jr., Sambandamurthy, V., Larsen, M., Tufariello, J., *et al.* (2002) Specialized transduction: an efficient method for generating marked and unmarked targeted gene disruptions in *Mycobacterium tuberculosis*, *M. bovis* BCG and *M. smegmatis*. *Microbiology* **148:** 3007-3017.

China, A. and Nagaraja, V. (2010) Purification of RNA polymerase from mycobacteria for optimized promoter-polymerase interactions. *Protein Expr Purif* **69:** 235-242.

Cole, S. T., Brosch, R., Parkhill, J., Garnier, T., Churcher, C., Harris, D., Gordon, S. V., Eiglmeier, K., Gas, S., Barry, C. E. 3rd, Tekaia, F., Badcock, K., Basham, D., Brown, D., Chillingworth, T., Connor, R., Davies, R., Devlin, K., Feltwell, T., Gentles, S., Hamlin, N., Holroyd, S., Hornsby, T., Jagels, K., Krogh, A., McLean, J., Moule, S., Murphy, L., Oliver, K., Osborne, J., Quail, M. A., Rajandream, M. A., Rogers, J., Rutter, S., Seeger, K., Skelton, J., Squares, R., Squares, S., Sulston, J. E., Taylor, K., Whitehead, S. and Barrell, B. G. (1998) Deciphering the biology of *Mycobacterium tuberculosis* from the complete genome sequence. *Nature* **393:** 537-544.

Gonzalez-y-Merchand, J. A., Colston, M. J. and Cox, R. A. (1996) The rRNA operons of *Mycobacterium smegmatis* and *Mycobacterium tuberculosis*: comparison of promoter elements and of neighbouring upstream genes. *Microbiology* **142 (Pt 3):** 667-674.

Kalate, R. N., Tambe, S. S. and Kulkarni, B. D. (2003) Artificial neural networks for prediction of mycobacterial promoter sequences. *Comput Biol Chem* **27:** 555-564.

Kumar, A., Deshane, J. S., Crossman, D. K., Bolisetty, S., Yan, B. S., Kramnik, I., Agarwal, A. and Steyn, A. J. (2008) Heme oxygenase-1-derived carbon monoxide induces the *Mycobacterium tuberculosis* dormancy regulon. *J Biol Chem* **283**: 18032-18039.

Pang, X., Vu, P., Byrd, T. F., Ghanny, S., Soteropoulos, P., Mukamolova, G. V., Wu, S., Samten, B. and Howard, S. T. (2007) Evidence for complex interactions of stress-associated regulons in an *mprAB* deletion mutant of *Mycobacterium tuberculosis*. *Microbiology* **153:** 1229-1242.

Rao, S. P., Alonso, S., Rand, L., Dick, T. and Pethe, K. (2008) The proton motive force is required for maintaining ATP homeostasis and viability of hypoxic, nonreplicating *Mycobacterium tuberculosis*. *Proc Natl Acad Sci USA* **105:** 11945-11950.

Saeed, A. I., Sharov, V., White, J., Li, J., Liang, W., Bhagabati, N., Braisted, J., Klapa, M., Currier, T., Thiagarajan, M., Sturn, A., Snuffin, M., Rezantsev, A., Popov, D., Ryltsov, A., Kostukovich, E., Borisovsky, I., Liu, Z., Vinsavich, A., Trush, V. and Quackenbush, J. (2003) TM4: a free, open-source system for microarray data management and analysis. *Biotechniques* **34:** 374-378.

Singh, A., Crossman, D. K., Mai, D., Guidry, L., Voskuil, M. I., Renfrow, M. B. and Steyn, A. J. (2009) *Mycobacterium tuberculosis* WhiB3 maintains redox homeostasis by regulating virulence lipid anabolism to modulate macrophage response. *PLoS Pathog* **5:** e1000545.

Smith, L. J., Stapleton, M. R., Fullstone, G. J., Crack, J. C., Thomson, A. J., Le Brun, N. E., Hunt, D. M., Harvey, E., Adinolfi, S., Buxton, R. S. and Green, J. (2010) *Mycobacterium tuberculosis* WhiB1 is an essential DNA-binding protein with a nitric oxide-sensitive iron-sulfur cluster. *Biochem J* **432:** 417-427.

Song, T., Song, S. E., Raman, S., Anaya, M. and Husson, R. N. (2008) Critical role of a single position in the -35 element for promoter recognition by *Mycobacterium tuberculosis* SigE and SigH. *J Bacteriol* **190:** 2227-2230.

Steyn, A. J., Collins, D. M., Hondalus, M. K., Jacobs, W. R. Jr., Kawakami, R. P. and Bloom, B. R. (2002) *Mycobacterium tuberculosis* WhiB3 interacts with RpoV to affect host survival but is dispensable for in vivo growth. *Proc Natl Acad Sci USA* **99:** 3147-3152.

Tusher, V. G., Tibshirani, R. and Chu, G. (2001) Significance analysis of microarrays applied to the ionizing radiation response. *Proc Natl Acad Sci USA* **98:** 5116-5121.

Verma, A., Kinger, A. K. and Tyagi, J. S. (1994) Functional analysis of transcription of the *Mycobacterium tuberculosis* 16S rDNA-encoding gene. *Gene* **148:** 113-118.

Zihlif, M., Catchpoole, D. R., Stewart, B. W. and Wakelin, L. P. (2010) Effects of DNA minor groove binding agents on global gene expression. *Cancer Genomics Proteomics* **7:** 323-330.
